# New insights into carbon metabolism in S*pathaspora passalidarum* for second-generation ethanol production

**DOI:** 10.3389/ffunb.2025.1657121

**Published:** 2025-09-19

**Authors:** Sofía Racca, Rodrigo J. Leonardi, Raúl N. Comelli

**Affiliations:** ^1^ Grupo de Procesos Biológicos en Ingeniería Ambiental (GPBIA), Facultad de Ingeniería y Ciencias Hídricas (FICH), Universidad Nacional del Litoral (UNL), Santa Fe, Argentina; ^2^ Agencia Nacional de Promoción Científica y Tecnológica (ANPCyT), Buenos Aires, Argentina; ^3^ Consejo Nacional de Investigaciones Científicas y Técnicas (CONICET), Buenos Aires, Argentina

**Keywords:** bioethanol, non-conventional yeast, *Spathaspora passalidarum*, yeast metabolism, fermentation, sugar metabolism, xylose

## Abstract

Bioethanol is a sustainable, low-impact energy source with the potential to reduce or even replace fossil fuel consumption. Second-generation (2G) bioethanol exploits lignocellulosic agro-industrial residues, contributing to circular economy strategies by valorizing these waste streams. However, conventional *Saccharomyces cerevisiae* strains are unable to efficiently metabolize the pentose sugars abundant in lignocellulose, prompting growing interest in non-conventional yeasts such as *Spathaspora passalidarum*. This species, recognized for its innate ability to assimilate pentoses, remains underexplored, particularly regarding its metabolic performance in mixed-sugar environments containing hexoses, pentoses, and disaccharides. Our results demonstrate that *S. passalidarum*’s xylose metabolism is strongly inhibited by pulses of hexoses such as glucose, galactose, and mannose, as well as by the disaccharide maltose. Notably, inhibition was also triggered by the non-metabolizable glucose analog 2-deoxyglucose (2DG), indicating that the regulatory signal originates during the early stages of glucose uptake into the cytosol rather than from downstream glycolytic pathways. In contrast, xylose metabolism was prioritized over fructose and sucrose. Furthermore, *S. passalidarum* was able to metabolize arabinose and glycerol, although these pathways favored biomass production through oxygen-dependent processes. Arabinose could be co-metabolized with xylose, but its assimilation was markedly suppressed in the presence of glucose. Collectively, these findings provide new insights into the metabolic regulation of *S. passalidarum* and highlight its potential role in the design of robust strategies for 2G bioethanol production.

## 1 Introduction

With the rising global energy demand and the environmental impact of fossil fuel use, biofuels have gained substantial attention as an alternative energy source (Jeswani et al., 2020). Among these, bioethanol is a liquid biofuel produced by yeasts and other microorganisms that convert carbohydrates into ethanol through fermentation. First-generation bioethanol (1G) is derived from grains, cereals, and sugarcane; however, its reliance on arable land and the resulting competition between food and fuel production have raised concerns. Second-generation bioethanol (2G) addresses these issues by utilizing lignocellulosic agricultural residues and sugar-rich industrial wastewaters as feedstock (Broda et al., 2022; Comelli, 2023).

Using lignocellulosic material as a feedstock, however, presents challenges. Lignocellulose is a complex structure comprising lignin, cellulose (hexoses: D-glucose), and hemicellulose (hexoses and pentoses: L-arabinose and D-xylose). To access these sugars, a physicochemical pretreatment with sulfuric acid and high temperatures is required, followed by enzymatic hydrolysis to release glucose from cellulose (Gil Rolón et al., 2023). Additionally, *Saccharomyces cerevisiae* strains commonly employed in bioethanol production lack the ability to efficiently utilize pentoses, limiting raw material conversion efficiency. Genetic modifications to enable pentose metabolism in *S. cerevisiae* have shown limited success, yielding low efficiencies under industrial conditions (Kuyper et al., 2005; Hahn-Hägerdal et al., 2007; Nevoigt, 2008; Lee et al., 2017; Endalur Gopinarayanan and Nair, 2019). Consequently, the study and evaluation of non-conventional yeasts with intrinsic pentose metabolism capabilities are of significant interest (Radecka et al., 2015; Geijer et al., 2022; Ndubuisi et al., 2023).

Bolzico et al (2024) evaluated xylose metabolism in non-conventional yeast strains and examined the role of oxygen in their fermentative performance, demonstrating promising results for *Spathaspora passalidarum* and *Scheffersomyces stipitis*, consistent with the findings from other studies (Kumar et al., 2009; da Cunha-Pereira et al., 2011; Cadete et al., 2012; Krahulec et al., 2012; Cadete and Rosa, 2018; Campos et al., 2022). A subsequent work by Guzmán et al (2024) further investigated *S. passalidarum* as a viable xylose-fermenting strain, assessing key industrial parameters such as the inoculum-to-substrate ratio, pH influence, and ethanol tolerance. This study reported a significant inhibitory effect of glucose on xylose metabolism, with pentoses only metabolized after glucose depletion, i.e., an important consideration when designing processes for mixed-sugar feedstocks. Moreover, *S. passalidarum* exhibited high ethanol sensitivity, with growth inhibition observed at concentrations of 20 g L^−1^ or higher, and was also sensitive to lignocellulosic pretreatment inhibitors like furfural and 5-hydroxymethylfurfural (HMF) (Hou and Yao, 2012; Soares et al., 2020).

For effective integration of *S. passalidarum* into an industrial 2G ethanol process, it is essential to strategically consider this metabolic profile to maximize yields while minimizing operational time. Given the variety of mixed-sugar residues available as feedstocks for bioethanol production (**Supplementary Figure S1**) (Friedman, 2013; Isla et al., 2013; He et al., 2014; Featherstone, 2015; Ikram et al., 2017; Comelli et al., 2018; Cruz et al., 2018; Palmonari et al., 2020; Ajala et al., 2021; Amaro Bittencourt et al., 2021; Mahmud and Anannya, 2021; Nair et al., 2022; Roukas and Kotzekidou, 2022; Zou and Chang, 2022; Gil Rolón et al., 2023) and the potential to combine sugar-rich industrial effluents with agro-industrial lignocellulosic residues (Comelli, 2023; de Oliveira Pereira et al., 2024), an expanded metabolic study of *S. passalidarum* across a broad carbohydrate spectrum, including hexoses, pentoses, and disaccharides, is necessary. Literature about sugar fermentation by *S. passalidarum* is still under development and mainly focused on glucose and xylose (Nguyen et al., 2006; Hou, 2012; Long et al., 2012; Rodrussamee et al., 2018; Ribeiro et al., 2021; Campos et al., 2022; Trichez et al., 2023; Bolzico et al., 2024; Guzmán et al., 2024; Saengphing et al., 2024), avoiding other monosaccharides and disaccharides (Cadete et al., 2009; Rodrussamee et al., 2018; Du et al., 2019; Farias and Maugeri-Filho, 2021; Pereira I de et al., 2021) (**Table 1**). In this study, we applied an innovative methodology to evaluate *S. passalidarum*’s metabolism of individual carbohydrates and to understand its adaptive response to sudden “pulses” of various sugars, including glucose, galactose, mannose, fructose, maltose, sucrose, lactose, and arabinose, on xylose consumption. We also extended our research on carbon metabolism by studying arabinose, an abundant but less frequently reported pentose in lignocellulosic biomass (Jacob et al., 2023), and glycerol, a low-cost carbon source derived from industry by-products (Abad and Turon, 2012; Kaur et al., 2020). These findings contribute to a deeper understanding of *S. passalidarum* and provide valuable insights for selecting or combining residues as feedstock in bioethanol production processes.

**Table 1:** Carbohydrate consumption and fermentation by *Spathaspora passalidarum*.

Sugar	Consumption	Fermentation	Reference
Glucose and xylose	Yes	Yes	(Nguyen et al., 2006; Long et al., 2012; Rodrussamee et al., 2018; Ribeiro et al., 2021; Campos et al., 2022; Trichez et al., 2023; Bolzico et al., 2024; Guzmán et al., 2024; Saengphing et al., 2024)
Fructose	Yes	Yes	(Farias and Maugeri-Filho, 2021; Pereira I de et al., 2021)
Arabinose	Yes	Arabitol—no ethanol detected	(Rodrussamee et al., 2018; Du et al., 2019; Farias and Maugeri-Filho, 2021)
Maltose	Yes	Yes	(Nguyen et al., 2006)
Manse	Yes	Yes	(Rodrussamee et al., 2018)
Sucrose	Yes	Pereira et al. reported ethanol production by *S. passalidarum* in reactors with 90 g L^−1^ of sucrose and intracellular sucrose hydrolysis. Nguyen reported sucrose consumption but no fermentation.	(Nguyen et al., 2006; Pereira I de et al., 2021)
Galactose	Yes	Yes	(Nguyen et al., 2006; Rodrussamee et al., 2018)
Lactose	No	No	(Nguyen et al., 2006; Cadete et al., 2009)

## 2 Materials and methods

### 2.1 Yeast strain, cell propagation, and culture media

The yeast strain *S. passalidarum* NRRL Y-27907 was used in this study. Long-term cell stocks were preserved in 30% (v/v) glycerol at −80°C. For working cultures, cells were reactivated by incubation in YPG medium (20 g L^−1^ of glucose, 5 g L^−1^ of yeast extract, 3 g L^−1^ of meat peptone) with chloramphenicol at 30°C for 24 h. A 5-µL aliquot of this culture was streaked onto solid YPG medium (1.5% agar) and incubated at 30°C until colony formation.

To prepare the inoculum for fermentation, a single colony was cultured in 100 mL of YPX medium (20 g L^−1^ of xylose, 10 g L^−1^ of yeast extract, 3 g L^−1^ of meat peptone) in a 500-mL Erlenmeyer flask at 30 °C with agitation at 150 rpm for 48 h. Following cultivation, cells were harvested by centrifugation at 4,500 rpm for 5 min, washed twice with sterile distilled water, and resuspended in 10 mL of water to prepare the final inoculum. The initial yeast concentration in each fermentation reactor was standardized to 1.0 g L^−1^.

### 2.2 Fermentation assays

Fermentations were conducted in biological duplicates or triplicates, in 100-mL glass reactors operating in batch mode. The reactors were maintained at 30 °C with constant agitation at 150 rpm, and 1-mL samples were taken every 4 to 6 h. At each sampling time, biomass growth, sugar consumption, and ethanol production were measured.

For single-sugar assays, a 60-mL medium containing 15 g L^−1^ of the sugar (xylose, glucose, galactose, mannose, fructose, sucrose, maltose, lactose), 7.5 g L^−1^ of yeast extract, and 3 g L^−1^ of meat peptone was used. Considering arabinose, a concentration of 10 g L^−1^ was chosen, representing a typical concentration present in lignocellulosic materials.

For xylose fermentation assays involving sugar pulses, 60 mL of YPX medium was used, and after 12 h of xylose fermentation, a sugar pulse was introduced by adding an exact volume of a sterile concentrated sugar solution (200 g L^−1^ of sugar and 100 g L^−1^ of yeast extract). This addition achieved a final pulse concentration of 15 g L^−1^ of sugar and 7.5 g L^−1^ of yeast extract. A control reactor with YPX but without any sugar pulse was also run. A scheme of sugar-pulse methodology is displayed in **Supplementary Figure S2**.

Arabinose and glycerol fermentations were performed in batch mode using 100-mL reactors with varying working volumes to adjust headspace: reactors with 70% headspace (30 mL of working volume, referred to as 70% HS), 40% headspace (60 mL of working volume), and 10% headspace (90 mL of working volume). The arabinose concentration was 10 g L^−1^, supplemented with 5 g L^−1^ of yeast extract and 3 g L^−1^ of meat peptone, while the glycerol concentration was 30 g L^−1,^ supplemented with 10 g L^−1^ of yeast extract and 3 g L^−1^ of meat peptone.

### 2.3 Analytical measurements

Samples were taken at different times and centrifuged at 4,500 rpm for 5 min. The supernatants were transferred to new tubes and stored at −20 °C until the appropriate determination.

#### 2.3.1 Biomass quantification

The cells were washed twice with distilled water and suspended in the starting volume. Optical density (OD) was measured at 600 nm using a VIS spectrophotometer (DR/2010, HACH, USA). Biomass quantification was determined using a specific curve made for the *S. passalidarum* strain, which allowed correlating DO_600_ and g L^−1^. The calibration curve was constructed based on the volatile suspended solids (VSS) determination method (Eaton et al., 1995; Comelli et al., 2016).

#### 2.3.2 Sugar, alcohol, and inhibitor quantification

Sugar, arabitol, xylitol, and acetate quantification were performed using high-performance liquid chromatography (UltiMate 3000 HPLC System, Thermo Fisher, USA) coupled to a refractive index detector (RID) (RefractoMax 520). Separation was conducted using the SH-1011 Column (Shodex, Japan) at a column temperature of 60 °C. The mobile phase consisted of 5 mM of H_2_SO_4_, with a flow rate of 0.6 mL min^−1^. Standard solutions of each analyte were used to determine retention time and to correlate chromatogram areas with concentration (g L^−1^) through a calibration curve.

#### 2.3.3 Glycerol quantification

Glycerol quantification was performed using the commercial colorimetric kit TG color GPO/PAP AA developed by Wienner Lab^®^ following protocol instructions. A standard solution of glycerol was employed to build standard curves.

#### 2.3.4 Ethanol quantification

Ethanol was quantified using a gas chromatograph (GC-2014 system, Shimadzu, USA) equipped with a flame ionization detector and the TR-Wax GC column (Thermo Fisher, USA).

### 2.4 Calculation of fermentation parameters

Fermentation parameters were calculated according to the methodology described by Guzmán et al (Guzmán et al., 2024). Biomass growth ΔX (g_biomass_ L^−1^) was quantified as the difference between final and initial biomass concentrations. Substrate consumption ΔS (percentage or (g_sugar_ L^−1^)) was calculated as the difference between initial and final substrate concentrations. Ethanol (p) yield (Y_p/s_, g_ethanol_/g_consumed sugar_) was calculated as the ratio of maximal ethanol production (g_ethanol_ L^−1^) ΔP (g_ethanol_ L^−1^) to sugar consumption ΔS (g_sugar_ L^−1^), whereas biomass (X) yield (Y_x/s_, g_biomass_/g_consumed sugar_) was calculated as the ratio of cellular growth ΔX (g_biomass_ L^−1^) to sugar consumption ΔS (g_sugar_ L^−1^). The specific growth rate (µ(x), h^−1^) of *S. passalidarum* was determined by the slope of the ln(X/X_0_) versus time, where X corresponds to final biomass and X_0_ corresponds to initial biomass. Substrate consumption rates (r(s), g_sugar_ (L h)^−1^), ethanol production rates (r(p), g_ethanol_ (L h)^−1^), and biomass growth rates (r(x), g_biomass_ (L h)^−1^) were calculated based on the slopes of the substrate (g L^−1^), ethanol (g L^−1^), and biomass (g L^−1^) versus time graphs, respectively. The *R* ^2^ values for each linear regression were found to be up to 0.95 ± 0.01.

### 2.4 Statistical analysis

The results obtained in triplicate were statistically analyzed using the ANOVA Tukey test (*n* = 3; *p* < 0.05) employing the Infostat^®^ software.

## 3 Results

### 3.1 *Spathaspora passalidarum* was able to ferment a wide range of carbohydrates

**Figure 1:**
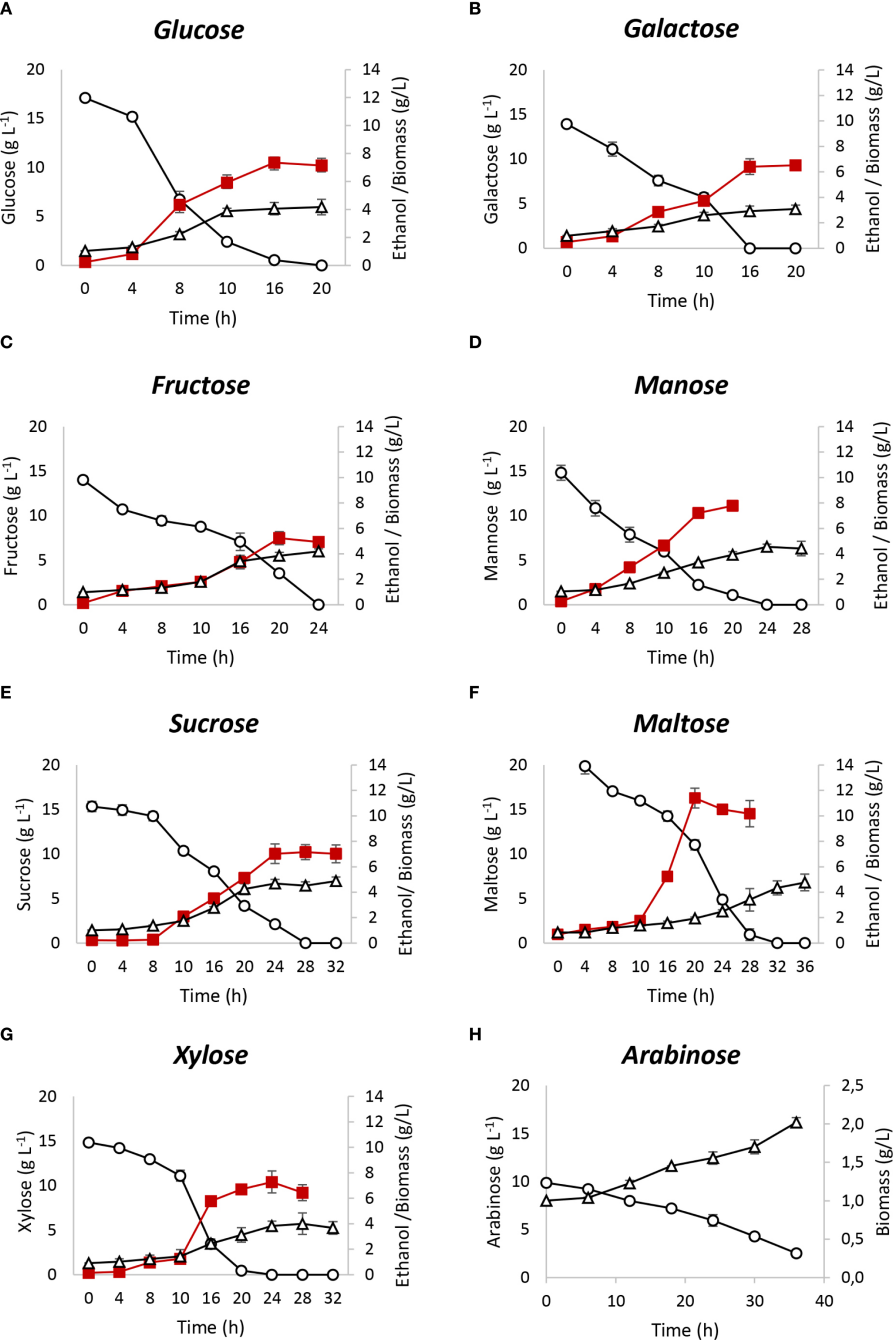
Fermentation performance of *Spathaspora passalidarum* using different carbohydrates. **(A)** glucose; **(B)** galactose; **(C)** fructose; **(D)** mannose; **(E)** sucrose; **(F)** maltose; **(G)** xylose; **(H)** arabinose. Circles indicate sugar consumption on the main axis, squares indicate ethanol production, and triangles indicate biomass production on the secondary axis.

To assess the fermentation performance of *S. passalidarum*, seven individual sugars, galactose, mannose, fructose, sucrose, maltose, lactose, and arabinose, were tested, with glucose and xylose used as controls. **Table 2** provides the stoichiometric and kinetic parameters for each fermentation, and **Figure 1** presents the performance results in terms of sugar consumption, biomass, and ethanol production.

**Table 2:** Average stoichiometric and kinetic parameters for *Spathaspora passalidarum* fermentations with individual sugars, conducted at 30 °C in batch reactors.

*Parameter*	*Xylose*	*Glucose*	*Galactose*	*Mannose*	*Fructose*	*Sucrose*	*Maltose*	*Arabinose*
r(x)	0.16 ± 0.002	0.14 ± 0.001	0.09 ± 0.001	0.15	0.22 ± 0.011	0.23 ± 0.002	0.06 ± 0.001	0.028 ± 0.001
r(s)	1.08 ± 0.01	1.06 ± 0.02	0.88 ± 0.02	0.87 ± 0.03	0.52 ± 0.02	0.61 ± 0.01	0.45/1.16 ± 0.01	0.2 ± 0.001
r(p)	0.53 ± 0.01	0.85 ± 0.01	0.45 ± 0.03	0.45 ± 0.01	0.3 ± 0.03	0.34 ± 0.03	0.09/0.93 ± 0.01	–
µ(x)	0.29 ± 0.02	0.18 ± 0.02	0.09 ± 0.01	0.07 ± 0.01	0.09 ± 0.01	0.09 ± 0.01	0.05 ± 0.001	0.031 ± 0.001
Y(x/s)	0.19 ± 0.03	0.26 ± 0.003	0.15 ± 0.01	0.24 ± 0.01	0.29 ± 0.02	0.24 ± 0.02	0.18 ± 0.01	0.14 ± 0.01
Y(p/s)	0.49 ± 0.04	0.43 ± 0.04	0.47 ± 0.05	0.49 ± 0.01	0.37 ± 0.03	0.46 ± 0.03	0.5 ± 0.03	–
ΔS (%)	100%	100%	100%	100%	100%	100%	100%	74% ± 1%
ΔX (g/L)	2.77 ± 0.49	3.9 ± 0.47	2.77 ± 0.42	3.57 ± 0.5	3.96 ± 0.34	3.86 ± 0.32	3.93 ± 0.26	1 ± 0.1
Time (h)	20	16	16	24	24	28	20	36

For hexoses, galactose and mannose yielded ethanol outputs similar to those of glucose and xylose, with ethanol yields of 0.47 and 0.49 g_ethano_l/g_consumed sugar_, respectively. Mannose fermentation produced a biomass yield (Yxs) of 0.24 g_biomass_/g_consumed sugar_, comparable to glucose and higher than xylose and galactose, which had Yxs values of 0.19 and 0.15 g_biomass_/g_consumed sugar_, respectively. Glucose and galactose were completely consumed within 16 h, while xylose and mannose required 20 and 24 h, respectively. Fructose fermentation showed a lower ethanol yield (Yps) of 0.37 g_ethano_l/g_consumed sugar_. Initial sugar consumption was slow (rs = 0.3 g_sugar_ (L h)^−1^ during the first 16 h), but accelerated to 0.88 g_sugar_ (L h)^−1^ between 16 and 24 h, resulting in complete fructose depletion. Among the disaccharides, lactose was not consumed by *S. passalidarum*, while sucrose and maltose reached maximum ethanol yields, with complete consumption in 28 h. Maltose fermentation showed a lower initial consumption rate (r(s) = 0.45 g_sugar_ (L h)^−1^) for the first 20 h, which increased significantly to 1.16 g_sugar_ (L h)^−1^ between 20 and 28 h. Considering sucrose, information available in the current literature does not provide clarity as to whether *S. passalidarum* is capable of fermenting this disaccharide. Our results show that this strain was able to consume and ferment sucrose with almost maximum ethanol yield. **Supplementary Figure S3** shows *S. passalidarum* fermentation of sucrose compared with a mixture of their monomers, glucose and fructose. Sucrose, glucose, and fructose consumption rates were independent of each other, with a significant difference in r(x). It can be observed that intracellular hydrolysis of sucrose with ethanol production reached the maximum yield.

For arabinose, no ethanol production was observed, as its metabolism was directed toward biomass in a slow process. After 36 h, only 74% of arabinose was consumed, with a biomass formation rate (rx) of 0.028 g_biomass_ (L h)^−1^. This aspect will be further detailed in Section 3.3.

### 3.2 Xylose consumption patterns in *Spathaspora passalidarum* are differentially delayed with the *pulse-of-sugar* methodology

The behavior of *S. passalidarum* has been studied using glucose and xylose as primary carbon sources (**Table 1**). However, additional studies are needed to assess its metabolic responses to mixed-sugar feedstocks. To investigate this, a pulse-of-sugar methodology was applied in which xylose metabolism was interrupted 12 h into fermentation by introducing a specific sugar pulse. This approach allows the assessment of *S. passalidarum*’s adaptation to abrupt metabolic shifts and the impact on fermentation performance.

**Figure 2:**
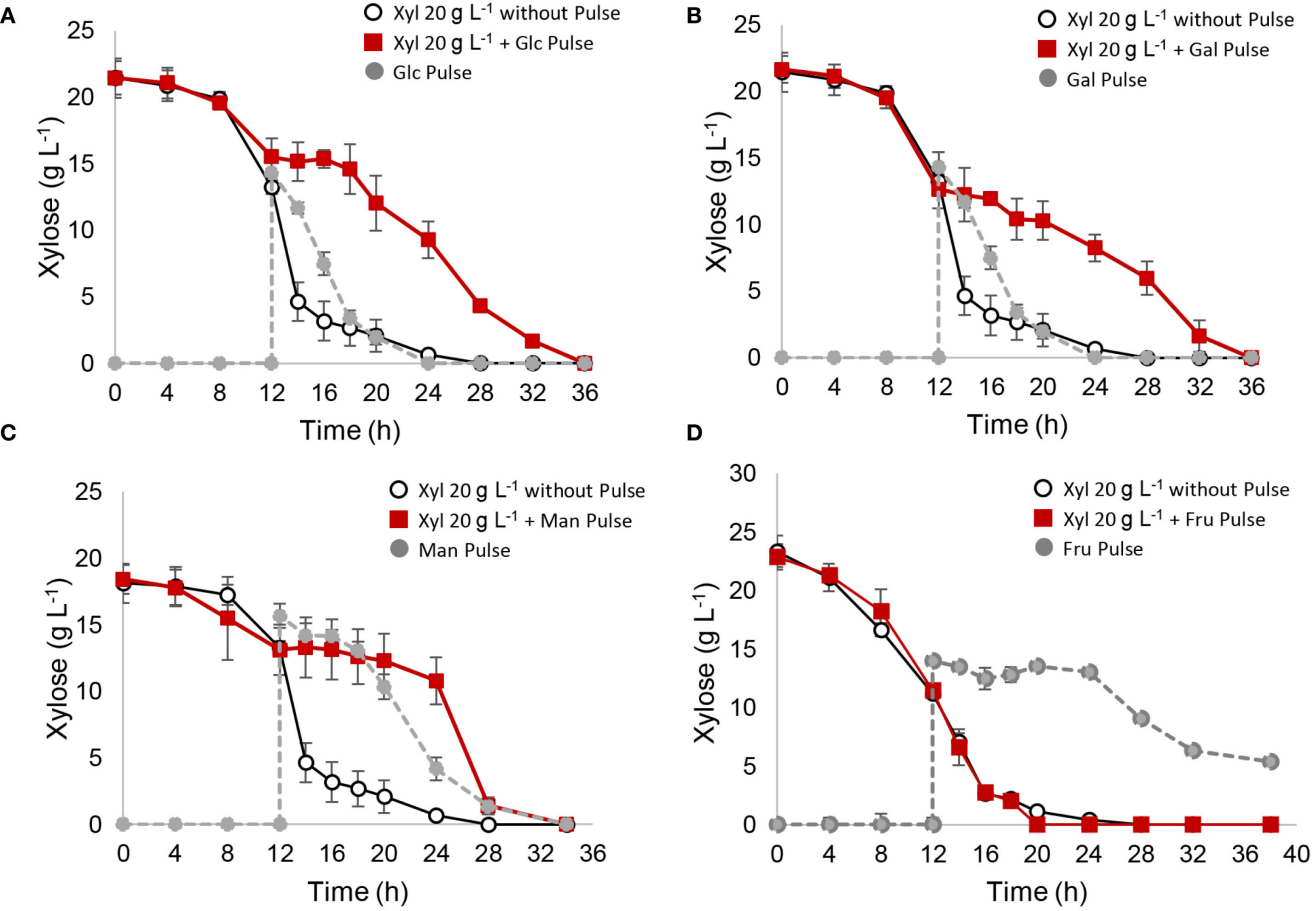
Xylose metabolism interrupted with different hexose pulses. After 12 h of xylose fermentation, the system was interrupted with a hexose pulse of 15 g L^−1^. A control reactor with YPX medium without pulse was used as control. **(A)** glucose pulse; **(B)** galactose pulse; **(C)** mannose pulse; **(D)** fructose pulse.

As shown in **Figure 2**, pulses of 15 g L⁻¹ glucose, galactose, or mannose significantly delayed xylose metabolism, with fructose being the only hexose that did not affect xylose consumption. To analyze these results, a relative consumption rate, r(s) (compared to the control with no pulse, where r(x) = 1), was calculated between 12 and 20 h to capture the period immediately after the pulse (**Table 3**). Glucose, galactose, and mannose pulses showed a significant reduction in relative r(s), immediately halting xylose consumption between 12 and 20 h, which resumed thereafter (**Figure 2** **;** **Table 3**). Regarding ethanol yields, pulses of glucose and galactose redirected metabolism toward ethanol, increasing the ethanol yield (Yps) to 0.50 and 0.46 g_ethanol_/g_consumed sugar_, respectively, compared to 0.41 g_ethanol_/g_consumed sugar_ in the control without a pulse (**Table 3**). In contrast, mannose and fructose pulses led to slightly lower yields, with Yps values of 0.39 and 0.35 g_ethanol_/g_consumed sugar_, respectively (**Table 3**). With fructose pulses, *S. passalidarum* prioritized pentose metabolism, initiating fructose consumption only once xylose was depleted. Biomass and ethanol quantification data for each pulse fermentation are shown in **Supplementary Figure S4**. The inhibitory effect of glucose on xylose metabolism was dose-dependent, as shown in **Supplementary Figure S5**, with greater inhibitory effects observed as glucose pulse concentration increased.

**Table 3:** Average kinetic and stoichiometric parameters for xylose interrupted with a sugar pulse after 12 h of fermentation.

*Parameter*	*Xyl (control)* without pulse	*Xyl + sugar pulse*						
		Glu	Gal	Fru	Man	Suc	Mal	Lac
*relative r(x)*	1.0 ± 0.09 (a)	**0**.**24 ± 0.02 (b)**	**0.17 ± 0.03 (b)**	1.05 ± 0.09 (a)	**0.06 ± 0.02 (b)**	0.98 ± 0.05 (a)	**0.15 ± 0.04 (b)**	0.97 ± 0.05 (a)
*Y(xs)*	0.13 ± 0.02	0.11 ± 0.01	0.08 ± 0.001	0.11 ± 0.01	0.07 ± 0.001	0.1 ± 0.01	0.1 ± 0.01	0.14 ± 0.02
*Y(ps)*	0.41 ± 0.02	0.5 ± 0.03	0.46 ± 0.03	0.39 ± 0.02	0.35 ± 0.01	0.33 ± 0.02	0.33 ± 0.02	0.41 ± 0.03

Relative r(x) (compared to control without pulse r(x)=1) was calculated between 12 and 20 h to consider the immediate period after pulse. Letters correspond to the ANOVA Tukey test; *n* = 3; *p* < 0.05. Different letters indicate a significant statistical difference, and they were highlighted in Bold. Biomass yield (Y(xs)) and ethanol yield (Y(ps)) are also shown.

**Figure 3:**
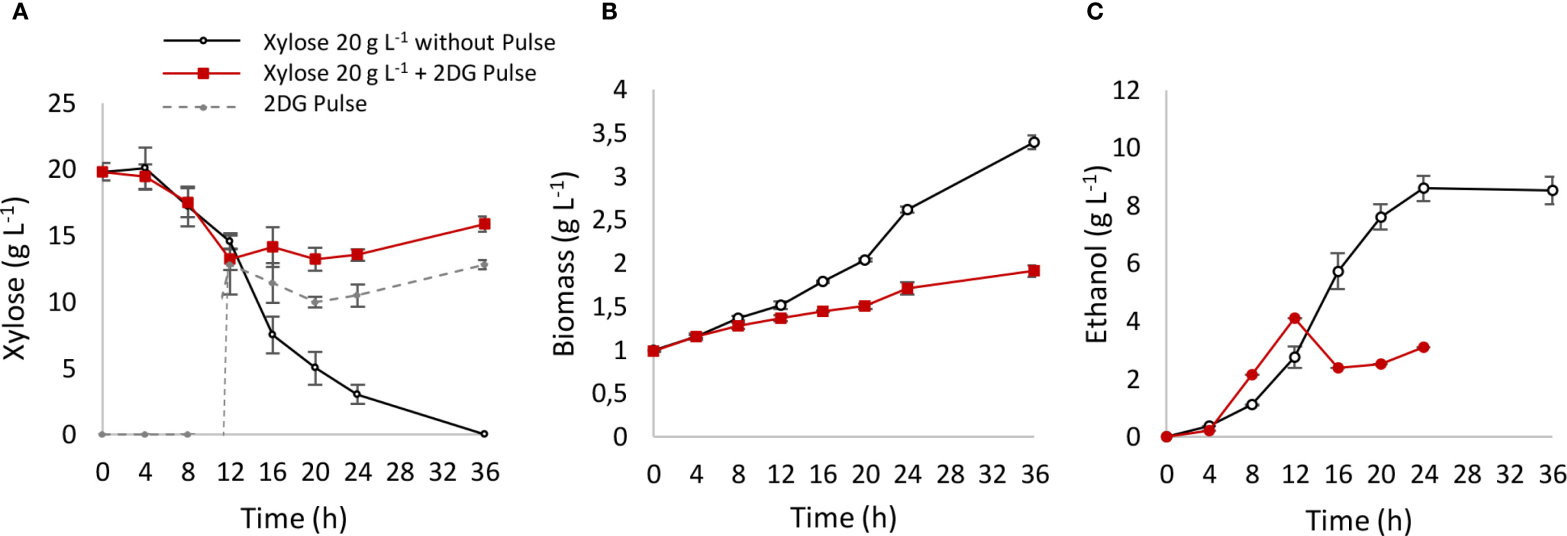
Xylose metabolism interrupted with a 2DG pulse. After 12 h of xylose fermentation, the system was interrupted with a pulse of 10 g L^−1^ of 2DG. A control reactor with YPX medium and without pulse was used as control. **(A)** xylose consumption pattern; **(B)** biomass production; **(C)** ethanol production.

To explore potential mechanisms underlying this behavior, fermentation was conducted in YPX medium, where xylose metabolism was interrupted by a pulse of 2-deoxyglucose (2DG), a non-metabolizable glucose analog (**Figure 3**). 2DG is capable of entering the cell and being phosphorylated by hexokinase, but it cannot be metabolized by the glycolytic pathway, aborting energetic metabolism. Additionally, 2DG produces a strong inhibitory signal, which completely halted xylose consumption (**Figure 3A**), resulting in a suppression of biomass (**Figure 3B**) and ethanol production (**Figure 3C**). This suggests that the inhibition of pentose metabolism may involve glucose recognition, transport, or cytosolic phosphorylation, potentially triggering a signaling cascade that halts pentose transport. Further studies will be needed to understand the role of hexose sensing in regulating pentose metabolism.

**Figure 4:**
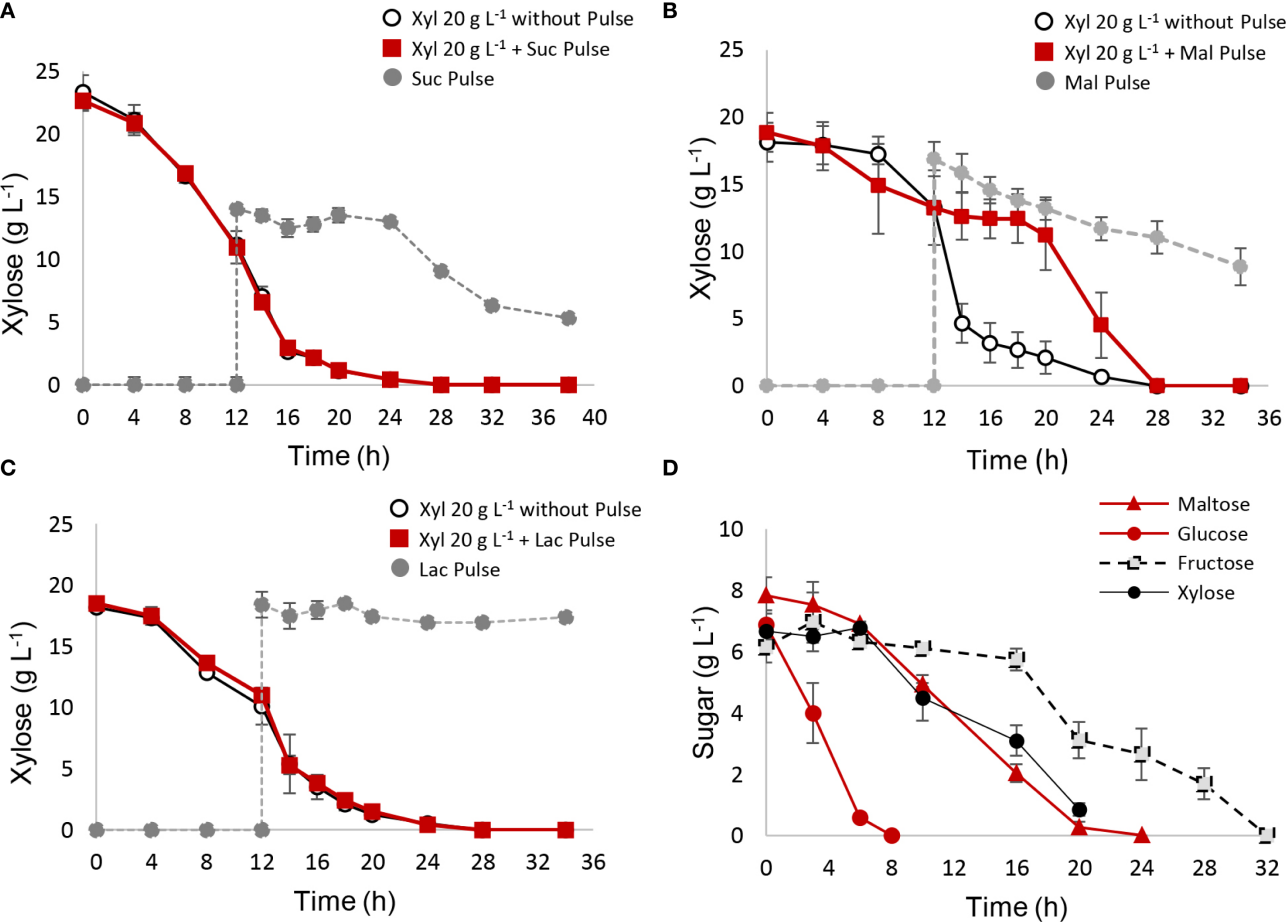
**(A–C)** Xylose metabolism interrupted with different disaccharide pulses. After 12 h of xylose fermentation, the system was interrupted with a pulse of 15 g L^−1^ of disaccharide. A control reactor with YPX medium and without pulse was used as control. **(A)** sucrose pulse; **(B)** maltose pulse; **(C)** lactose pulse. **(D)** Fermentation carried out with four-sugar media consisting of glucose, maltose, xylose, and fructose in equal ratios.

Pulse assays were also conducted using disaccharides (**Figures 4A–C**). Among the tested disaccharides (sucrose, lactose, and maltose), only maltose inhibited xylose consumption between 12 and 20 h, reducing the xylose consumption rate (r(s)) during this period (**Table 3**). Sucrose consumption did not begin until xylose was fully depleted. Additionally, reactors with sucrose or maltose pulses exhibited lower biomass and ethanol yields (**Table 3**), while lactose pulses had no impact on xylose metabolism or yields. Ethanol and biomass quantifications for each disaccharide pulse are shown in **Supplementary Figure S6**.

In addition to pulse strategy, it is interesting to evaluate *S. passalidarum* performance in the presence of mixed composition media, where different types of sugars were present from the initial time. Fermentation was carried out in the presence of 30 g L^−1^ sugar media, consisting of equal amounts of glucose, fructose, xylose, and maltose (**Figure 4D**). Metabolic behavior was predictable, and *S. passalidarum* showed a high preference for glucose, which was quickly depleted after 6–8 h. Maltose was consumed more slowly during 24 h, possibly due to the time required for disaccharide hydrolysis. The presence of glucose and maltose inhibited the initial xylose consumption, which has a lag phase of 8 h and a total time of 24 h to be depleted. Finally, fructose metabolism required 32 h, being the last sugar to be fully consumed. Mixture fermentation was able to produce a ΔX of 3.92 ± 0.42 g L^−1^ and 12.43 ± 0.12 g L^−1^ of ethanol, with a Y(xs) of 0.130 ± 0.001 and Y(ps) of 0.41 ± 0.01. An additional assay was performed changing sugar ratios, using a xylose concentration of 15 g L^−1^ and 5 g L^−1^ of glucose, fructose, and maltose. The results are displayed in **Supplementary Figure S7**. We can conclude that xylose exhibited 12 h of lag phase and a significant increase in its consumption rate after 12 and 24 h, after glucose and maltose depletion, respectively. Maltose and xylose are depleted at the same time, after 28 h of fermentation, and fructose was the last sugar to be consumed, in times longer than 32 h. Ethanol yield presented a value of 0.5 ± 0.02, while biomass generation was lower due to the high amount of xylose compared with other sugars (Yxs = 0.110 ± 0.005).

### 3.3 Arabinose and glycerol metabolism in *Spathaspora passalidarum*

**Figure 5:**
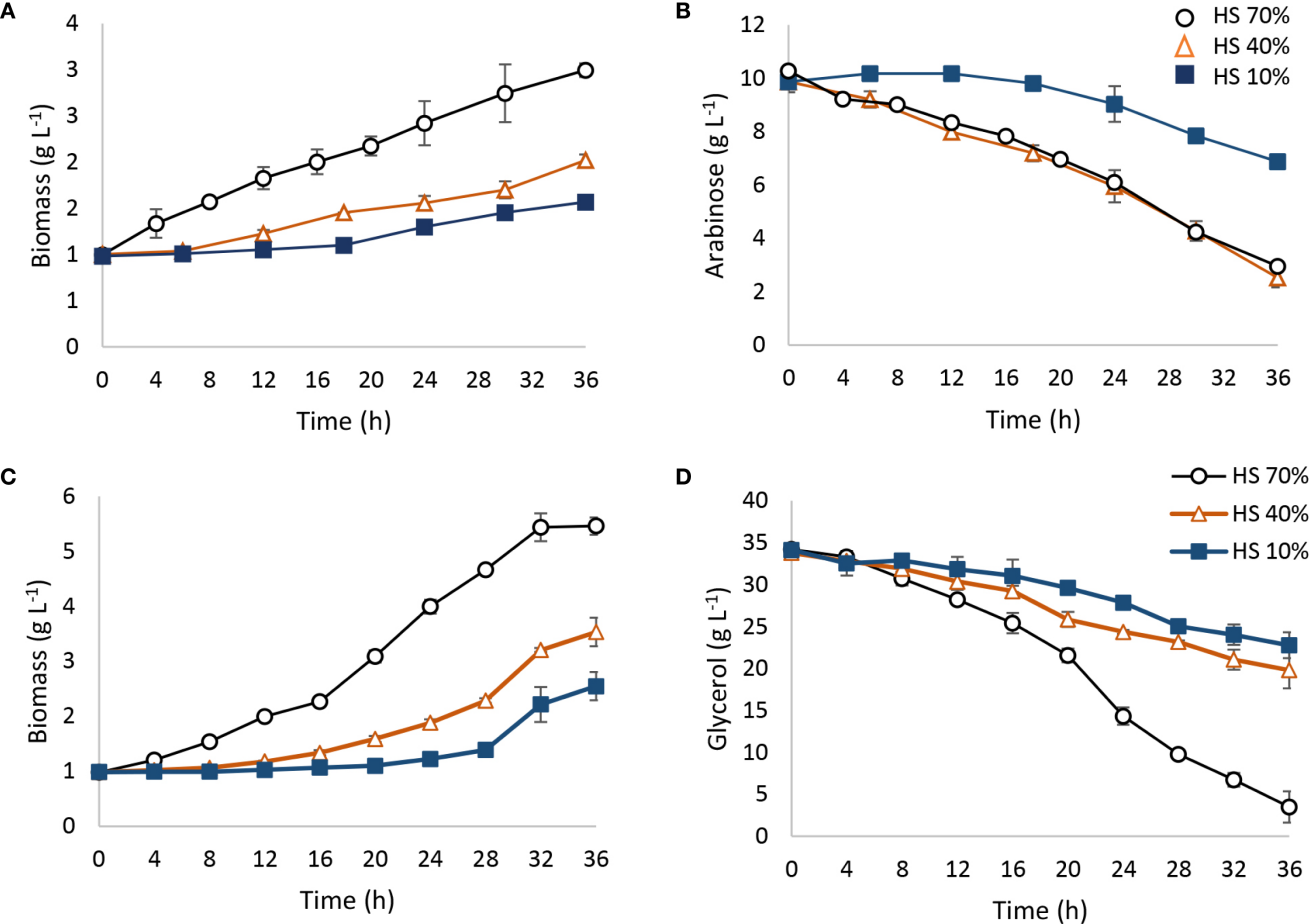
Arabinose and glycerol fermentation by *Spathaspora passalidarum*. Fermentations were carried out in 100-mL batch reactors with different working volumes: 30, 60, and 90 mL (70% HS, 40% HS, and 10% HS) depending on oxygen requirements. A temperature of 30°C and constant agitation of 150 rpm were employed. **(A, B)** Arabinose metabolism; **(C, D)** glycerol metabolism.

*Spathaspora passalidarum* was able to metabolize arabinose; however, 36 h of fermentation did not allow for complete sugar depletion (**Figures 5A, B**). Arabinose consumption rate was strongly influenced by oxygen availability, represented as % HS (**Figure 5B**). Under 70% HS and 40% HS conditions, approximately 80% of the arabinose was consumed, with an r(s) of 0.204 g_arabinose_ (L h)^−1^ (**Table 4**), while under 10% HS, only 30% of the arabinose was metabolized, with an r(s) of 0.083 g_arabinose_ (L h)^−1^ (**Table 4**). No ethanol production was detected in any aeration condition, although arabitol concentrations of 2.87 and 1.1 g L⁻¹ were measured in the 70% HS and 40% HS conditions, yielding Y(ps) values of 0.35 and 0.14 g_arabitol_/g_consumed arabinose_, respectively. Acetate was undetected at 36 h, but glycerol concentrations between 0.83 and 0.9 g_glycerol_ L⁻¹ were observed across all tested conditions. These results suggest that in *S. passalidarum*, arabinose metabolism is directed toward biomass and arabitol production in a highly oxygen-dependent process, but does not lead to ethanol, possibly due to challenges in NAD^+^ regeneration. **Table 4** presents the stoichiometric and kinetic parameters for arabinose metabolism under each condition.

**Table 4:** Stoichiometric and kinetic parameters for arabinose and glycerol fermentation by *Spathaspora passalidarum*.

Parameter	Arabinose			Glycerol		
	70% HS	40% HS	10% HS	70% HS	40% HS	10% HS
*Y(xs)*	0.27 ± 0.02	0.14 ± 0.01	0.19 ± 0.01	0.15 ± 0.01	0.18 ± 0.01	0.14 ± 0.01
*r(x)*	0.054 ± 0.002	0.028 ± 0.001	0.017 ± 0.001	0.124 ± 0.002	0.071 ± 0.001	0.040 ± 0.001
*µ(x)*	0.029 ± 0.001	0.031 ± 0.001	0.024 ± 0.001	0.057 ± 0.001	0.036 ± 0.001	0.019 ± 0.001
*r(s)*	0.204 ± 0.010	0.204 ± 0.01	0.083 ± 0.002	0.85 ± 0.03	0.38 ± 0.02	0.31 ± 0.02

Fermentations were performed in 100-mL batch reactors with varying working volumes: 30, 60, and 90 mL (70% HS, 40% HS, and 10% HS) to assess different oxygen requirements. Conditions included a temperature of 30 °C and constant agitation at 150 rpm.

On the other hand, *S. passalidarum* was able to use glycerol as a carbon source, redirecting the energy flux to biomass with no ethanol production (**Figures 5C, D**). Biomass generation was also dependent on % HS in reactors, showing a high demand for aeration (**Figure 5C**). The maximum amount of glycerol consumption was obtained in 70% HS reactors, while both decreased directly with the HS percentage (**Figure 5D**). Stoichiometric and kinetic parameters for glycerol metabolism in each condition are displayed in **Table 4**.

**Figure 6:**
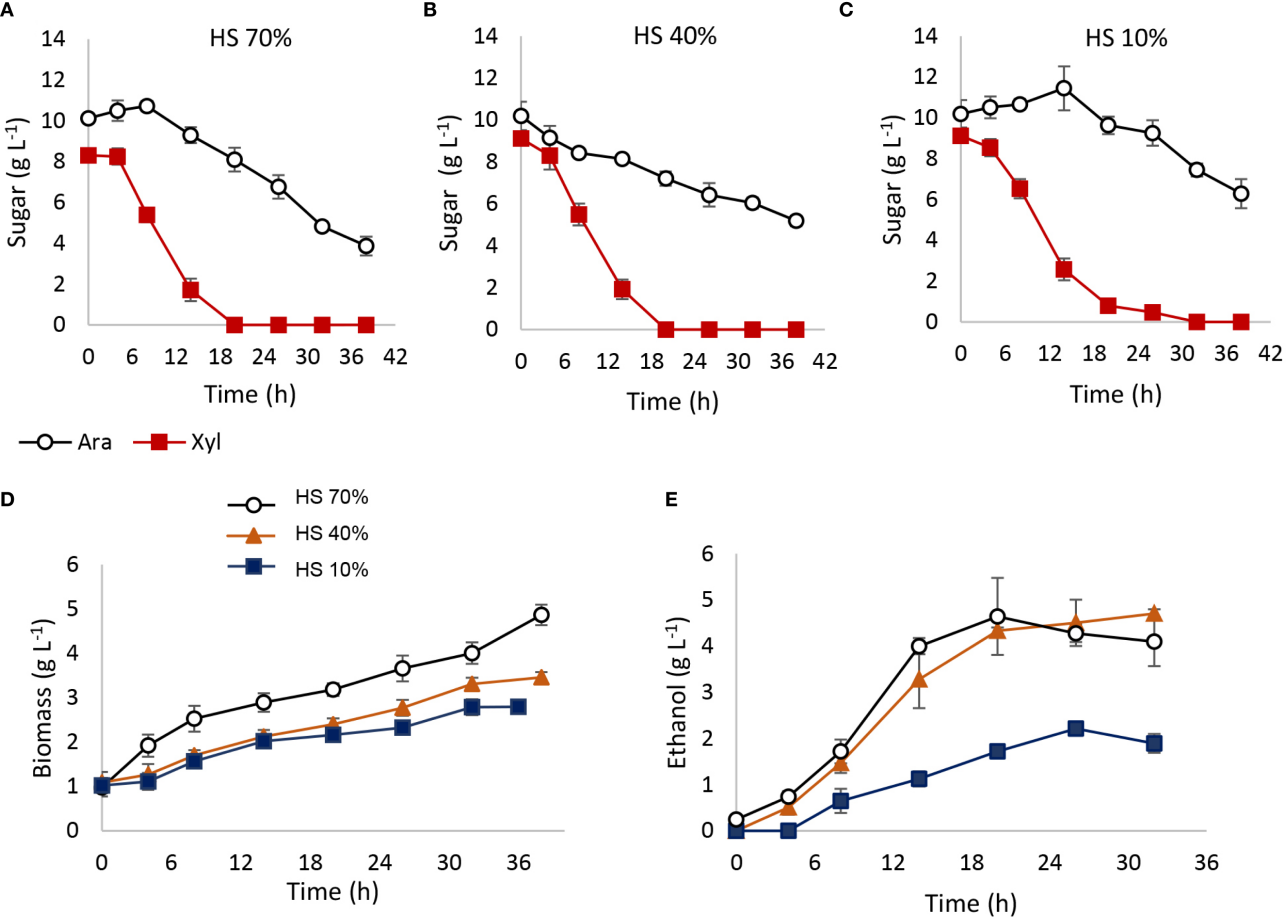
Co-fermentations with xylose and arabinose. Fermentations were carried out in 100-mL batch reactors with different working volumes: 30, 60, and 90 mL (70% HS, 40% HS, and 10% HS) depending on oxygen requirements. **(A–C)** Arabinose and xylose quantification in 70% HS, 40% HS, and 10% HS reactors. **(D)** Biomass production; **(E)** ethanol production.

Co-fermentation experiments with 10 g L⁻¹ of xylose and 10 g L⁻¹ of arabinose demonstrated that both pentoses could be consumed simultaneously (**Figure 6**). Over a 42-h fermentation period, xylose was completely depleted within 18 h in reactors with 70% and 40% HS (**Figures 6A, B**), while 30 h was required in the 10% HS condition (**Figure 6C**). In terms of arabinose consumption, 42 h of fermentation led to 60%, 50%, and 40% depletion in reactors with 70%, 40%, and 10% HS, respectively (**Figures 6A–C**). Biomass production was enhanced by higher oxygen availability (**Figure 6D**), while ethanol production reached only 4.5 g L⁻¹, attributable solely to xylose fermentation (**Figure 6E**). Arabitol, glycerol, and acetate were absent in samples from reactors with 40% and 10% HS, though 0.53 g L⁻¹ of arabitol and 0.7 g L⁻¹ of glycerol were detected in 70% HS reactors. An additional assay demonstrates that an arabinose pulse does not inhibit xylose metabolism (**Supplementary Figure S8**).

**Figure 7:**
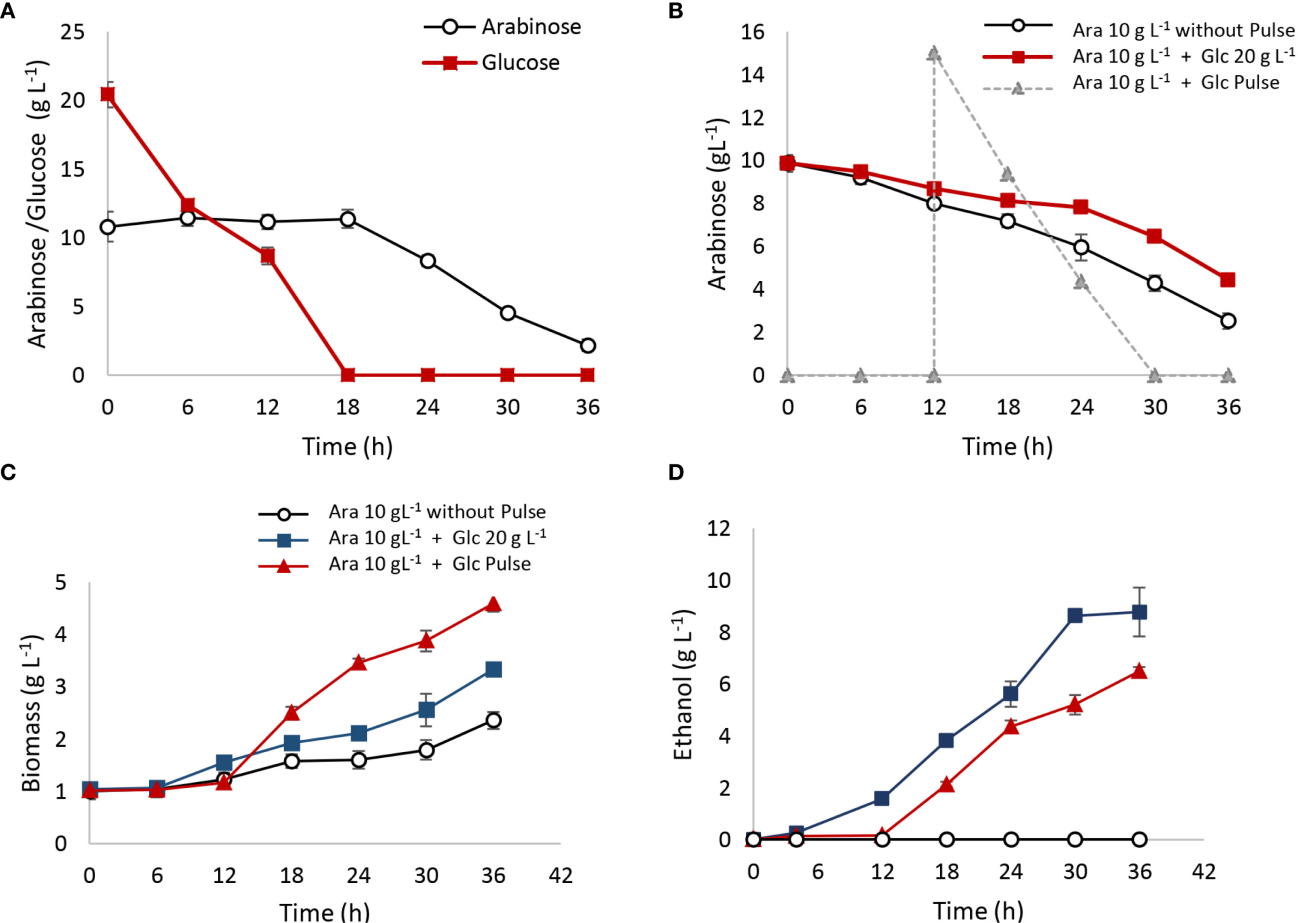
Co-fermentations with arabinose and glucose. Fermentations were carried out in 100-mL batch reactors with a working volume of 60 mL (40% HS). **(A)** Mixture of 10 g L^−1^ of arabinose and 20 g L^−1^ of glucose since the initial time; **(B)** arabinose 10 g L^−1^ was interrupted with a 15 g L^−1^ of glucose pulse after 12 h of fermentation; **(C)** biomass production; **(D)** ethanol production.

Co-fermentation trials with 20 g L⁻¹ of glucose and 10 g L⁻¹ of arabinose were conducted in 100-mL batch reactors with a working volume of 60 mL (40% HS) over 36 h, with results detailed in **Figure 7**. As shown in **Figure 7A**, arabinose was not consumed until glucose was completely depleted. Biomass production increased to ΔX = 2 g L⁻¹, compared to ΔX = 1 g L⁻¹ observed in the 10 g L⁻¹ arabinose control (**Figure 7C**). Ethanol production reached 8 g_ethanol_ L⁻¹, consistent with the glucose fermentation yield (Yps glucose = 0.4 g_ethanol_/g_consumed sugar_) (**Figure 7D**).

An additional assay evaluated the effect of a 15 g L⁻¹ glucose pulse on arabinose metabolism after 12 h of fermentation (**Figure 7B**). The glucose pulse delayed arabinose consumption. In the control with 10 g L⁻¹ of arabinose and no pulse, 80% of the arabinose was consumed after 36 h. In reactors with the glucose pulse, however, only 60% of arabinose was metabolized within the same period, indicating an inhibitory effect of glucose on arabinose metabolism. Biomass and ethanol production in the reactors with the glucose pulse were ΔX = 3 g_biomass_ L⁻¹ and 6 g_ethanol_ L⁻¹, respectively, compared to ΔX = 1 g_biomass_ L⁻¹ and no ethanol detected in the arabinose-only control (**Figures 7C, D**).

## 4 Discussion

*Spathaspora passalidarum* has been proposed as a promising non-conventional yeast for 2G ethanol production due to its ability to metabolize xylose from lignocellulosic materials and sugar-rich industrial effluents efficiently (Cadete and Rosa, 2018; Campos et al., 2022; Bolzico et al., 2024). This capacity enables greater utilization of the hemicellulosic fraction, increasing ethanol yield. However, integrating *S. passalidarum* into a 2G process poses challenges, notably the need for an expanded understanding of its metabolic behavior in mixed-sugar streams. For example, glucose and other carbohydrates have been shown to inhibit xylose metabolism, a phenomenon known as carbon catabolite repression (Subtil and Boles, 2012; Lane et al., 2018; Rodrussamee et al., 2018; Simpson-Lavy and Kupiec, 2019). This effect, common among yeasts and other microorganisms, leads to the prioritization of energetically favorable sugars like glucose over secondary sugars, as described by Zhao et al (Zhao et al., 2020).

Our results demonstrated a dose-dependent inhibition of xylose metabolism by glucose. A sudden pulse of glucose—or other hexoses such as galactose or mannose—was sufficient to temporarily interrupt xylose consumption, indicating that the metabolism of these sugars is prioritized over that of pentoses in this strain. The observation that a pulse of 2DG, a non−metabolizable (but phosphorylatable) glucose analog, also delayed xylose utilization, suggests that the underlying mechanism is triggered by glucose sensing or its immediate cytosolic entry, rather than full metabolic processing. A similar approach was previously reported by Ribeiro et al (Ribeiro et al., 2021); however, in their design, 2DG was present from the start of the culture, resulting in an extended lag phase followed by eventual xylose consumption. Consequently, the authors did not report a complete inhibition of pentose utilization. In contrast, the pulse−based strategy employed in the present study allowed us to capture the immediate adaptive response of the yeast to a sudden sugar shift, providing a more precise view of the transient regulatory phenomenon that occurs during ongoing xylose fermentation.

Although molecular signaling pathways in *S. passalidarum* remain poorly characterized, insights can be drawn from the well−studied glucose-signaling network in *S. cerevisiae* (Kayikci and Nielsen, 2015; Persson et al., 2022). Glucose−mediated catabolite repression primarily involves three interconnected routes: i) the HXK2/Snf1/Mig1 pathway represses the transcription of genes required for the utilization of alternative carbon sources. The glucose hexokinase HXK2 allows glucose sensing, and it is involved in Mig1 phosphorylation and in their nucleocytoplasmic distribution (Ahuatzi et al., 2007); ii) the Rgt2/Snf3 signaling cascade, which regulates the expression of HXT glucose transporters and promotes the degradation of other sugar permeases; and iii) the cAMP/PKA pathway, which stimulates fermentative growth under glucose while suppressing stress responses and respiratory metabolism. The step of glucose phosphorylation by a hexokinase could be involved in pentose repression mediated by glucose and 2DG, and the use of other non-phosphorylatable analogs like 4DG or 6DG to obtain additional information is necessary. Recently, it was reported that an overexpression of Snf3 improves signaling in the presence of xylose, suggesting that this receptor is involved in extracellular pentose sensing in *S. cerevisiae* (Bolzico et al., 2025).

While elucidating the specific molecular mechanism in *S. passalidarum* was beyond the scope of this work, our pulse−based experimental design provides a promising platform for future transcriptomic and regulatory analyses, particularly through RNA−seq, to identify the transcriptional responses and key regulators that govern hexose–pentose co−utilization in this species.

Regarding the experiments with disaccharides, it is noteworthy that interrupting a xylose culture with a maltose pulse was also able to delay pentose consumption. Subtil and Boles (2012) reported similar inhibitory effects of glucose on both xylose and arabinose in recombinant *S. cerevisiae* strains, with pentose metabolism resuming only after glucose depletion. They proposed a competitive inhibition mechanism where hexoses, due to their higher affinity for transporters, outcompete pentoses. Additionally, in their co-fermentation studies of xylose and maltose, they observed that maltose partially inhibited xylose consumption. This inhibition suggested that intracellular accumulation of D-glucose could also disrupt pentose metabolism. Our experiments are consistent with these findings: a maltose pulse effectively interrupted xylose metabolism, indicating that *S. passalidarum* prioritized maltose over xylose. Interestingly, the inhibitory effect could be partially mitigated by overexpressing the hexose transporters HXT7 and Gal2.

We could hypothesize that the accumulation and phosphorylation of glucose in the cytosol, immediately after its uptake, may represent the key step in signal transduction to inhibit pentose metabolism. Nevertheless, sucrose, which is hydrolyzed to glucose and fructose, does not trigger the same effect. This could suggest the presence of specific signaling pathways mediated by membrane receptors or transporters. Transporters, known as maltose permeases, mediate maltose transport in *S. cerevisiae*. Once inside the cytosol, maltose is cleaved into two glucose monomers by an enzyme with α-glucosidase activity (maltase) (Brondijk et al., 2001). In the case of sucrose, this disaccharide is mainly hydrolyzed in the periplasm into fructose and glucose through the action of an invertase (β-fructofuranosidase). Both fructose and glucose subsequently enter the cytosol via HXT-family transporters (Marques et al., 2016). Non-conventional yeasts such as *Ogataea polymorpha* utilize intracellular α-glucosidases for hydrolyzing sucrose, relying on specific permeases for sugar uptake. In the case of *S. passalidarum*, this strain showed intracellular sucrose hydrolysis and a low uptake rate, reported by Pereira et al (Pereira I de et al., 2021).

We observed that the inhibitory effect of glucose on xylose is dose-dependent, suggesting that the levels of glucose generated through disaccharide hydrolysis, as well as the rate of hydrolysis and subsequent cytosolic glucose accumulation, could influence the magnitude of the response. In *S. passalidarum*, sucrose exhibited a longer lag phase than maltose. This indicates that it could be more metabolically prepared for the consumption of maltose, hence its ability to delay the metabolism of xylose. Additional studies are necessary to fully elucidate these mechanisms. It is also noteworthy that the inhibitory effect of glucose can be reversed at higher xylose concentrations. Soares et al (2020) observed this phenomenon in co-fermentation trials with xylose and glucose, using lignocellulosic hydrolysates of soybean hulls with high xylose and low glucose concentrations. A xylose-glucose ratio of 3:1 reported in this work using a four-sugar medium cannot avoid glucose and maltose repression on xylose metabolism.

When designing a 2G ethanol production process from lignocellulosic material or mixed composition raw materials, the inhibitory effect of hexoses on xylose must be considered. It becomes evident that *S. passalidarum* prioritizes glucose as its primary carbon source, a behavior that has been reported (Guzmán et al., 2024). In lignocellulosic feedstock, pentoses are metabolized only after glucose depletion. In a mixed sugar environment—such as that potentially arising from the integration of 1G/2G processes, composed of industrial and agro-industrial effluents—fermentation times are expected to vary depending on the sugar ratios. Xylose metabolism accelerated as glucose and maltose were depleted, whereas fructose exhibited the longest lag phase and a lower consumption rate, and thus would likely represent the main contributor to the delayed fermentation when using *S. passalidarum*. *Spathaspora passalidarum* prioritized xylose over fructose and sucrose, and curiously, their consumption only started after pentose depletion, extending fermentation time and resulting in a lower ethanol yield. This point is important due to the abundance of these sugars in some industrial wastewaters that could be combined with lignocellulosic residues.

In this context, several hypotheses can be proposed to optimize fermentation times. First, employing initial or sequential mixed inocula that combine conventional *Saccharomyces* with non-conventional yeasts could be advantageous. Various industrial *S. cerevisiae* strains are highly efficient at rapidly consuming glucose, even at high concentrations, and can also contribute to the consumption of sucrose and maltose. Conversely, *S. passalidarum* could play a key role in the fermentation of pentoses, as well as hexoses and disaccharides.

However, despite the promise of this combination, a critical bottleneck threatens overall yield: the clear inhibition of *S. passalidarum* by ethanol accumulation or by the presence of inhibitors like acetate, furfural, and HMF derived from lignocellulose pretreatment. Recent studies have reported encouraging results using laboratory-adapted native strains, which exhibited improved tolerance to inhibitors and even co-fermentation capacity of glucose and xylose (González-Ramos et al., 2016; Pacheco et al., 2021; Fernandes et al., 2023; Trichez et al., 2023). Additionally, detoxification methods can be applied to remove inhibitors from sugar hydrolysates.

The generation of inhibitory compounds in lignocellulosic hydrolysates is inherently unavoidable, as the high temperature and acidic pretreatments typically employed promote the release of acetate, furfural, and HMF. The extent of inhibitor formation is also strongly influenced by the type of lignocellulosic feedstock. Consequently, a variety of detoxification strategies have been developed to mitigate their impact on fermentation performance (Parawira and Tekere, 2011). Among physical–chemical approaches, modified chitosan–chitin hybrid aerogels have demonstrated high removal efficiencies for HMF (85.1%) and furfural (99%), without compromising fermentable sugars, whereas activated carbon has shown comparable performance (Liu et al., 2024). Ion-exchange resins, particularly under alkaline conditions, selectively remove organic acids and phenolics, improving hydrolysate fermentability (Nilvebrant et al., 2001). Electrodialysis represents another effective method for the removal of acetate and phenolic compounds, although its efficiency toward furfural and HMF remains limited (Lee et al., 2013; Lee et al., 2014). Enzymatic approaches, including the use of laccases and other ligninolytic enzymes, have been shown to degrade phenolics and furan derivatives, simultaneously enhancing fermentability and reducing overall toxicity (Fernández-Sandoval et al., 2024). In addition, chemical neutralization, sulfite addition, solvent extraction, and alkaline treatments have been explored, although these methods may alter the sugar composition of the hydrolysate (Palmqvist and Hahn-hägerdal, 2000). Biological detoxification using specific microorganisms, such as *Cupriavidus basilensis* HMF14 and *Bordetella* sp. BTIITR, is particularly attractive as these strains selectively degrade furfural, HMF, and acetate without consuming sugars (Wierckx et al., 2010; Singh et al., 2019). Despite these advances, the high ethanol sensitivity of *S. passalidarum* remains a critical bottleneck, as the desired product itself exerts inhibitory effects. Therefore, strategies based on adaptive evolution or the development of genetically improved variants with enhanced ethanol tolerance emerge as a crucial step toward the efficient industrial exploitation of this yeast.

The molecular mechanisms governing pentose transport and consumption in non-conventional yeast strains remain less understood. Among yeasts with effective xylose metabolism, such as *S. passalidarum* and *Sc. stipitis*, xylose is taken up by both low- and high-affinity transporters. Low-affinity transporters facilitate the diffusion of xylose and glucose and are related to the *S. cerevisiae* HXT transporter family, with Hxt4p, Hxt5p, Hxt7p, and Gal2p mediating xylose uptake, albeit with significantly lower affinity compared to glucose (Leandro et al., 2009). In contrast, high-affinity transporters, functioning as xylose/H^+^ symporters, are crucial under low xylose conditions. While the identification and characterization of xylose transporters in non-conventional yeasts are still in early stages, some advances have been made. Examples include the low-affinity transporter Sut1–3 from *Sc. stipitis* CBS5774 and *Candida intermedia*’s GXF1 and GXS1 transporters, which facilitate diffusion and symport, respectively (Leandro et al., 2009).

Regarding arabinose, the second most abundant pentose, there are still no functional studies on arabinose transporters in yeasts. However, its uptake seems to be independent of xylose and glucose and is likely mediated by specific transporters. The first system facilitates diffusion with low affinity for arabinose, which has been characterized in *Pichia (Meyerozyma) guilliermondii* and *Candida arabinofermentans* (Fonseca et al., 2007). This kind of transport is induced by high arabinose concentrations. The authors also reported a higher-affinity arabinose transport system mediated by an arabinose/H^+^ symporter, which plays a major role under low concentrations of arabinose. Both transport systems were repressed by glucose. According to the literature, *S. cerevisiae* can poorly incorporate arabinose by facilitating the diffusion of hexose transporters Gal2, Hxt5, and Hxt7. Other transporters with higher affinity have been identified in *Neurospora crassa* (LAT-1), *Myceliophthora thermophila*, *Penicillium chrysogenum* (AraT), and *Sc. stipitis* (Ye et al., 2019). In the fungal pathway, L-arabinose is first converted into its corresponding polyol by the enzyme *arabitol reductase* (AR), and L-arabitol is subsequently oxidized to L-xylulose in a reaction mediated by *L-arabinose dehydrogenase* (LAD). The utilization of L-arabinose requires an additional reduction by *L-xylulose reductase* (LXR), converting L-xylulose into xylitol, the common intermediate for both arabinose and xylose catabolic pathways. Xylitol is then converted into D-xylulose by *xylitol dehydrogenase* (XDH), which is phosphorylated to xylulose-5P by a kinase (XK) (Fonseca et al., 2007). This final molecule can serve as an intermediate in the pentose phosphate pathway to produce ethanol. In filamentous fungi, L-arabinose and D-xylose reductases prefer NADPH as a cofactor, while sugar alcohol dehydrogenases strictly depend on NAD^+^. The fungal L-arabinose pathway is not redox balanced, and the cell’s capacity to regenerate NAD^+^ under low-oxygen conditions is limited, which can lead to arabitol accumulation (Fonseca et al., 2007).

Few studies on yeasts have identified some strains, such as *C. arabinofermentans*, *C. tropicalis*, *C. shehatae*, *M. guilliermondii*, *Pachysolen tannophilus*, *Sc. stipitis*, and *Torulopsis sonorensis* as arabinose consumers under aerobic conditions (Kurtzman and Dien, 1998; Fonseca et al., 2007; Ye et al., 2019). Most of them direct arabinose toward biomass production. However, the three strains of *Candida* spp. have been reported to produce traces of ethanol (Y(ps) = 0.18 g_ethanol_/g_consumed arabinose_) in media with high arabinose concentrations, as the pathway accumulates arabitol and minimal xylitol. Additionally, *Sc. stipitis* has been reported to produce 0.24 (g_arabitol_/g_consumed sugar_) and 0.15 (g_ethanol_/g_consumed sugar_) (Ye et al., 2019). *Rhodosporidium toruloides* can also accumulate D-arabitol from xylose (Jagtap and Rao, 2017). *Zygosaccharomyces rouxii* and *Z. siamensis* have shown a high capacity to transform glucose into arabitol, although they can also produce ethanol and glycerol as by-products (Saha et al., 2007; Iwata et al., 2023). Some genetically modified strains have been reported to express genes from the arabinose pathway of filamentous fungi in combination with xylose pathway genes (Ye et al., 2019). For instance, an *S. cerevisiae* strain expressing AR, XDH, and XK genes, along with the *Sc. stipitis* genes XYL1, XYL2, and XYL3 necessary for arabinose transport and metabolism, has been reported. Furthermore, this strain was subsequently modified by adding the LAD and LXR genes from *Trichoderma reesei* and *Ambroziosyma monospora*, respectively, but ethanol yields obtained remained at very low levels. Expanding the scope of research to include other non-conventional yeasts like *S. passalidarum* will expand our knowledge about arabinose metabolism and increase the reservoir of genes involved.

Our results showed that *S. passalidarum* can utilize arabinose for biomass production in a highly oxygen-dependent process but was unable to produce ethanol from this substrate. No significant ethanol production was detected, likely due to the inability to regenerate the reducing power required by the enzymes in this pathway. On the other hand, yields of 0.35 g_arabitol_/g_arabinose_ were obtained in 70% HS reactors after 36 h of fermentation and 80% arabinose consumption. Arabitol yields between 0.43 and 0.53 g_arabitol_/g_arabinose_ were reported for *Sc. stipitis* and *M. guilliermondii* and higher values for a few *Candida* species (0.6–0.8 g_arabitol_/g_arabinose_) (Kordowska-Wiater, 2015). Data collected until now propose that arabinose seems not able to ferment to ethanol by any wild or recombinant strain, wasting that fraction of lignocellulose.

The use of different cofactors by arabinose pathway enzymes generates a redox imbalance, especially under oxygen-limited conditions, where NAD^+^ regeneration is insufficient. As a result, the conversion of L-arabitol to L-xylulose becomes inefficient, and L-arabitol accumulates intracellularly (Fonseca et al., 2007). The accumulation of arabitol may also serve as a mechanism to regenerate NAD^+^, allowing metabolism to continue even if ethanol is not produced efficiently (Fonseca et al., 2007). Arabitol accumulation often occurs at the expense of ethanol production under certain conditions, such as osmotic stress or nitrogen limitation (Kordowska-Wiater, 2015; Yang et al., 2021). In some cases, arabitol and ethanol production can occur simultaneously, but generally, there is metabolic competition between the two pathways. The literature indicates that arabitol can act as a redox buffer, preventing oxidative damage (Sánchez-Fresneda et al., 2013), an osmoprotectant, or a carbon reserve that is not immediately used in the most demanding energy production pathways (Blakley and Spencer, 1962).

In the case of glycerol, similar results were obtained, where *S. passalidarum* was able to grow but not ferment using this carbon source. This topic could be interesting for inoculum production. The ability of yeast to metabolize glycerol holds significant economic value, as it enables the valorization of glycerol, an abundant and low-cost by-product of the biodiesel process (Abad and Turon, 2012). Utilizing glycerol as a carbon source can reduce the production costs of yeast biomass and enhance process sustainability. Furthermore, it supports the development of circular economy strategies by converting industrial waste into high-value bioproducts. A saturated culture of *S. passalidarum* obtained after 48 h of growth in glycerol was successfully used as inoculum for a subsequent fermentation in xylose, obtaining the same ethanol yield and kinetic and stoichiometric parameters compared with a 48-h inoculum produced with xylose as a carbon source (data not shown).

## 5 Conclusion

This study aimed to expand our knowledge on the metabolic behavior of *S. passalidarum* to assess its potential for 2G ethanol production. Due to its inherent ability to ferment xylose, *S. passalidarum* presents an interesting alternative for bioethanol production. However, understanding its performance in mixed-sugar streams is crucial for optimizing ethanol yields. Our findings show that xylose metabolism in *S. passalidarum* is strongly inhibited by hexoses such as glucose, mannose, and galactose, as well as by the disaccharide maltose. In contrast, *S. passalidarum* prioritized xylose consumption over fructose and sucrose, which were only metabolized after xylose depletion. This delayed their consumption, extending fermentation time and slightly reducing ethanol yield. Additionally, while *S. passalidarum* could metabolize arabinose, this conversion favored biomass generation over ethanol, indicating that this pentose would be underutilized in the feedstock.

These results provide strategic insights for designing an efficient 2G ethanol production process, enabling the effective use of sugars in raw materials and minimizing operational time. *Spathaspora passalidarum* shows potential as part of a combined fermentation system alongside *S. cerevisiae*, harnessing the complementary metabolic strengths of each yeast to achieve sequential and complete sugar utilization. However, it is necessary to focus on overcoming the high sensitivity of *S. passalidarum* to inhibitors and ethanol. The inhibitor removal process and laboratory-adaptive evolution are pointed out as the most promising alternatives to solve this limitation, although it is still under development. In conclusion, this work significantly enhances the metabolic understanding of *S. passalidarum*, encompassing fermentation dynamics across a range of sugars and sugar combinations, which have been underexplored in previous studies.

## Data Availability

The original contributions presented in the study are included in the article/**Supplementary Material**. Further inquiries can be directed to the corresponding author.

## References

[B1] Abad S. Turon X. (2012). Valorization of biodiesel derived glycerol as a carbon source to obtain added-value metabolites: Focus on polyunsaturated fatty acids. Biotechnol. Adv. 30, 733–741. doi: 10.1016/j.bioteChadv.2012.01.002, PMID: 22261015

[B2] Ahuatzi D. Riera A. Pélaez R. Herrero P. Moreno F. (2007). Hxk2 regulates the phosphorylation state of mig1 and therefore its nucleocytoplasmic distribution*. J. Biol. Chem. 282, 4485–4493. doi: 10.1074/jbc.M606854200, PMID: 17178716

[B3] Ajala E. O. Ighalo J. O. Ajala M. A. Adeniyi A. G. Ayanshola A. M. (2021). Sugarcane bagasse: a biomass sufficiently applied for improving global energy, environment and economic sustainability. Bioresour Bioprocess 8, 87. doi: 10.1186/s40643-021-00440-z, PMID: 38650274 PMC10991612

[B4] Amaro Bittencourt G. Porto de Souza Vandenberghe L. Valladares-Diestra K. Wedderhoff Herrmann L. Fátima Murawski de Mello A. Sarmiento Vásquez Z.. (2021). Soybean hulls as carbohydrate feedstock for medium to high-value biomolecule production in biorefineries: A review. Bioresour Technol. 339, 125594. doi: 10.1016/j.biortech.2021.125594, PMID: 34311407

[B5] Blakley E. R. Spencer J. F. T. (1962). Studies on the formation of d-arabitol by osmophilic yeasts. Can. J. Biochem. Physiol. 40, 1737–1748. doi: 10.1139/o62-192, PMID: 13971477

[B6] Bolzico B. C. Persson V. C. Comelli R. N. Gorwa-Grauslund M. (2025). Glucose receptor deletion and engineering: impact on xylose sensing and utilization in Saccharomyces cerevisiae. FEMS Yeast Res., foaf040. doi: 10.1093/femsyr/foaf040, PMID: 40728911 PMC12359139

[B7] Bolzico B. C. Racca S. Khawam J. N. Leonardi R. J. Tomassi A. H. Benzzo M. T.. (2024). Exploring xylose metabolism in non-conventional yeasts: kinetic characterization and product accumulation under different aeration conditions. J. Ind. Microbiol. Biotechnol. 51, kuae023. doi: 10.1093/jimb/kuae023, PMID: 38936832 PMC11247345

[B8] Broda M. Yelle D. J. Serwańska K. (2022). Bioethanol production from lignocellulosic biomass-challenges and solutions. Molecules 27, 8717. doi: 10.3390/molecules27248717, PMID: 36557852 PMC9785513

[B9] Brondijk T. H. Konings W. N. Poolman B. (2001). Regulation of maltose transport in Saccharomyces cerevisiae. Arch. Microbiol. 176, 96–105. doi: 10.1007/s002030100300, PMID: 11479708

[B10] Cadete R. M. Melo M. A. Dussán K. J. Rodrigues R. C. L. B. Silva S. S. Zilli J. E.. (2012). Diversity and physiological characterization of D-xylose-fermenting yeasts isolated from the Brazilian Amazonian Forest. PLoS One 7, e43135. doi: 10.1371/journal.pone.0043135, PMID: 22912807 PMC3418277

[B11] Cadete R. M. Rosa C. A. (2018). The yeasts of the genus Spathaspora: potential candidates for second-generation biofuel production. Yeast 35, 191–199. doi: 10.1002/yea.3279, PMID: 28892565

[B12] Cadete R. M. Santos R. O. Melo M. A. Mouro A. Gonçalves D. L. Stambuk B. U.. (2009). Spathaspora arborariae sp. nov., a d-xylose-fermenting yeast species isolated from rotting wood in Brazil. FEMS Yeast Res. 9, 1338–1342. doi: 10.1111/j.1567-1364.2009.00582.x, PMID: 19840117

[B13] Campos V. J. Ribeiro L. E. Albuini F. M. de Castro A. G. Fontes P. P. da Silveira W. B.. (2022). Physiological comparisons among Spathaspora passalidarum, Spathaspora arborariae, and Scheffersomyces stipitis reveal the bottlenecks for their use in the production of second-generation ethanol. Braz. J. Microbiol. 53, 977–990. doi: 10.1007/s42770-022-00693-6, PMID: 35174461 PMC9151973

[B14] Comelli R. N. (2023). “Chapter 4: Agro-industrial wastewaters as feedstocks: new research on bioethanol production,” in Agroindustrial research updates (Nova Science Publishers Inc, Hauppauge, New York). Available online at: https://novapublishers.com/shop/agricultural-research-updates-volume-43/. Prathamesh Gorawala and Srushti Mandhatri.

[B15] Comelli R. N. Seluy L. G. Benzzo M. T. Isla M. A. (2018). Combined Utilization of Agro-Industrial Wastewaters for Non-lignocellulosic Second-Generation Bioethanol Production Springer Science and Business Media B.V. Netherlands. Waste Biomass Valor 11, 265–275. doi: 10.1007/s12649-018-0391-x

[B16] Comelli R. N. Seluy L. G. Isla M. A. (2016). Optimization of a low-cost defined medium for alcoholic fermentation–a case study for potential application in bioethanol production from industrial wastewaters. N Biotechnol. 33, 107–115. doi: 10.1016/j.nbt.2015.09.001, PMID: 26391675

[B17] Cruz M. G. Bastos R. Pinto M. Ferreira J. M. Santos J. F. Wessel D. F.. (2018). Waste mitigation: From an effluent of apple juice concentrate industry to a valuable ingredient for food and feed applications. J. Cleaner Production 193, 652–660. doi: 10.1016/j.jclepro.2018.05.109

[B18] da Cunha-Pereira F. Hickert L. R. Sehnem N. T. de Souza-Cruz P. B. Rosa C. A. Ayub M. A. Z. (2011). Conversion of sugars present in rice hull hydrolysates into ethanol by Spathaspora arborariae, Saccharomyces cerevisiae, and their co-fermentations. Bioresour Technol. 102, 4218–4225. doi: 10.1016/j.biortech.2010.12.060, PMID: 21220201

[B19] de Oliveira Pereira I. Dos Santos Â. A. Guimarães N. C. Lima C. S. Zanella E. Matsushika A.. (2024). First- and second-generation integrated process for bioethanol production: Fermentation of molasses diluted with hemicellulose hydrolysate by recombinant Saccharomyces cerevisiae. Biotechnol. Bioeng 121, 1314–1324. doi: 10.1002/bit.28648, PMID: 38178588

[B20] Du C. Li Y. Zhao X. Pei X. Yuan W. Bai F.. (2019). The production of ethanol from lignocellulosic biomass by Kluyveromyces marxianus CICC 1727–5 and Spathaspora passalidarum ATCC MYA-4345. Appl. Microbiol. Biotechnol. 103, 2845–2855. doi: 10.1007/s00253-019-09625-1, PMID: 30706114

[B21] Eaton A. Clesceri L. Greenberg A. E. (1995). Standard methods for the examination of water and wastewater. Available online at: https://www.semanticscholar.org/paper/Standard-methods-for-the-examination-of-water-and-Eaton-Clesceri/9b605f9905a4e484592bd130376b668dfd9c880a (Accessed October 6, 2023). M.A.H F.

[B22] Endalur Gopinarayanan V. Nair N. U. (2019). Pentose metabolism in saccharomyces cerevisiae: the need to engineer global regulatory systems. Biotechnol. J. 14, e1800364. doi: 10.1002/biot.201800364, PMID: 30171750 PMC6452637

[B23] Farias D. Maugeri-Filho F. (2021). Sequential fed batch extractive fermentation for enhanced bioethanol production using recycled *Spathaspora passalidarum* and mixed sugar composition. Fuel 288, 119673. doi: 10.1016/j.fuel.2020.119673

[B24] Featherstone S. (2015). “8 - Ingredients used in the preparation of canned foods,” in *A Complete Course in Canning and Related Processes (Fourteenth Edition).* Woodhead Publishing Series in Food Science, Technology and Nutrition. Ed. Featherstone S. (Woodhead Publishing, Oxford), 147–211. doi: 10.1016/B978-0-85709-678-4.00008-7

[B25] Fernandes T. Osório C. Sousa M. J. Franco-Duarte R. (2023). Contributions of adaptive laboratory evolution towards the enhancement of the biotechnological potential of non-conventional yeast species. J. Fungi 9, 186. doi: 10.3390/jof9020186, PMID: 36836301 PMC9964053

[B26] Fernández-Sandoval M. T. García A. Teymennet-Ramírez K. V. Arenas-Olivares D. Y. Martínez-Morales F. Trejo-Hernández M. R. (2024). Removal of phenolic inhibitors from lignocellulose hydrolysates using laccases for the production of fuels and chemicals. Biotechnol. Prog. 40, e3406. doi: 10.1002/btpr.3406, PMID: 37964692

[B27] Fonseca C. Romão R. Rodrigues de Sousa H. Hahn-Hägerdal B. (2007). Spencer-Martins I. L-Arabinose transport and catabolism in yeast. FEBS J. 274, 3589–3600. doi: 10.1111/j.1742-4658.2007.05892.x, PMID: 17627668

[B28] Friedman M. (2013). Rice brans, rice bran oils, and rice hulls: composition, food and industrial uses, and bioactivities in humans, animals, and cells. J. Agric. Food Chem. 61, 10626–10641. doi: 10.1021/jf403635v, PMID: 24175575

[B29] Geijer C. Ledesma-Amaro R. Tomás-Pejó E. (2022). Unraveling the potential of non-conventional yeasts in biotechnology. FEMS Yeast Res. 22, foab071. doi: 10.1093/femsyr/foab071, PMID: 35040953 PMC8793870

[B30] Gil Rolón M. Leonardi R. J. Bolzico B. C. Seluy L. G. Benzzo M. T. Comelli R. N. (2023). Multi-response optimization of thermochemical pretreatment of soybean hulls for 2G-bioethanol production. Fermentation 9, 454. doi: 10.3390/fermentation9050454

[B31] González-Ramos D. Gorter de Vries A. R. Grijseels S. S. van Berkum M. C. Swinnen S. van den Broek M.. (2016). A new laboratory evolution approach to select for constitutive acetic acid tolerance in Saccharomyces cerevisiae and identification of causal mutations. Biotechnol. Biofuels 9, 173. doi: 10.1186/s13068-016-0583-1, PMID: 27525042 PMC4983051

[B32] Guzmán V. M. Leonardi R. J. Racca S. Comelli R. N. (2024). Assessing Process Conditions on Xylose Fermentation in Spathaspora passalidarum: Effects of pH, Substrate-to-Inoculum Ratio, Temperature, and Initial Ethanol Concentration. Curr. Microbiol. 81, 448. doi: 10.1007/s00284-024-03976-3, PMID: 39508833

[B33] Hahn-Hägerdal B. Karhumaa K. Fonseca C. Spencer-Martins I. Gorwa-Grauslund M. F. (2007). Towards industrial pentose-fermenting yeast strains. Appl. Microbiol. Biotechnol. 74, 937–953. doi: 10.1007/s00253-006-0827-2, PMID: 17294186

[B34] He Y. Dong J. Yin H. Zhao Y. Chen R. Wan X.. (2014). Wort composition and its impact on the flavour-active higher alcohol and ester formation of beer – a review. J. Institute Brewing 120, 157–163. doi: 10.1002/jib.145

[B35] Hou X. (2012). Anaerobic xylose fermentation by Spathaspora passalidarum. Appl. Microbiol. Biotechnol. 94, 205–214. doi: 10.1007/s00253-011-3694-4, PMID: 22124720

[B36] Hou X. Yao S. (2012). Improved inhibitor tolerance in xylose-fermenting yeast Spathaspora passalidarum by mutagenesis and protoplast fusion. Appl. Microbiol. Biotechnol. 93, 2591–2601. doi: 10.1007/s00253-011-3693-5, PMID: 22116630

[B37] Ikram S. Huang L. Zhang H. Wang J. Yin M. (2017). Composition and nutrient value proposition of brewers spent grain. J. Food Sci. 82, 2232–2242. doi: 10.1111/1750-3841.13794, PMID: 28833108

[B38] Isla M. A. Comelli R. N. Seluy L. G. (2013). Wastewater from the soft drinks industry as a source for bioethanol production. Bioresour Technol. 136, 140–147. doi: 10.1016/j.biortech.2013.02.089, PMID: 23567674

[B39] Iwata K. Maeda M. Kashiwagi Y. Maehashi K. Yoshikawa J. (2023). Isolation of Zygosaccharomyces siamensis kiy1 as a novel arabitol-producing yeast and its arabitol production. AMB Express 13. doi: 10.1186/s13568-023-01581-4, PMID: 37452923 PMC10349796

[B40] Jacob S. Dilshani A. Rishivanthi S. Khaitan P. Vamsidhar A. Rajeswari G.. (2023). Lignocellulose-derived arabinose for energy and chemicals synthesis through microbial cell factories: A review. Processes 11, 1516. doi: 10.3390/pr11051516

[B41] Jagtap S. Rao C. V. (2017). Production of d-arabitol from d-xylose by the oleaginous yeast Rhodosporidium toruloides IFO0880. Appl. Microbiol. Biotechnol. 102, 143–151. doi: 10.1007/s00253-017-8581-1, PMID: 29127468

[B42] Jeswani H. K. Chilvers A. Azapagic A. (2020). Environmental sustainability of biofuels: a review. Proc. Math Phys. Eng. Sci. 476, 20200351. doi: 10.1098/rspa.2020.0351, PMID: 33363439 PMC7735313

[B43] Kaur J. Sarma A. K. Jha M. K. Gera P. (2020). Valorisation of crude glycerol to value-added products: Perspectives of process technology, economics and environmental issues. Biotechnol. Rep. 27, e00487. doi: 10.1016/j.btre.2020.e00487, PMID: 32642454 PMC7334398

[B44] Kayikci Ö. Nielsen J. (2015). Glucose repression in Saccharomyces cerevisiae. FEMS Yeast Res. 15, fov068. doi: 10.1093/femsyr/fov068, PMID: 26205245 PMC4629793

[B45] Kordowska-Wiater M. (2015). Production of arabitol by yeasts: current status and future prospects. J. Appl. Microbiol. 119, 303–314. doi: 10.1111/jam.12807, PMID: 25809659

[B46] Krahulec S. Kratzer R. Longus K. Nidetzky B. (2012). Comparison of Scheffersomyces stipitis strains CBS 5773 and CBS 6054 with regard to their xylose metabolism: implications for xylose fermentation. Microbiologyopen 1, 64–70. doi: 10.1002/mbo3.5, PMID: 22950013 PMC3426399

[B47] Kumar A. Singh L. K. Ghosh S. (2009). Bioconversion of lignocellulosic fraction of water-hyacinth (Eichhornia crassipes) hemicellulose acid hydrolysate to ethanol by Pichia stipitis. Bioresour Technol. 100, 3293–3297. doi: 10.1016/j.biortech.2009.02.023, PMID: 19297151

[B48] Kurtzman C. P. Dien B. S. (1998). Candida arabinofermentans, a new L-arabinose fermenting yeast. Antonie Van Leeuwenhoek 74, 237–243. doi: 10.1023/a:1001799607871, PMID: 10081583

[B49] Kuyper M. Hartog M. M. P. Toirkens M. J. Almering M. J. H. Winkler A. A. van Dijken J. P.. (2005). Metabolic engineering of a xylose-isomerase-expressing Saccharomyces cerevisiae strain for rapid anaerobic xylose fermentation. FEMS Yeast Res. 5, 399–409. doi: 10.1016/j.femsyr.2004.09.010, PMID: 15691745

[B50] Lane S. Xu H. Oh E. J. Kim H. Lesmana A. Jeong D.. (2018). Glucose repression can be alleviated by reducing glucose phosphorylation rate in Saccharomyces cerevisiae. Sci. Rep. 8, 2613. doi: 10.1038/s41598-018-20804-4, PMID: 29422502 PMC5805702

[B51] Leandro M. J. Fonseca C. Gonçalves P. (2009). Hexose and pentose transport in ascomycetous yeasts: an overview. FEMS Yeast Res. 9, 511–525. doi: 10.1111/j.1567-1364.2009.00509.x, PMID: 19459982

[B52] Lee Y.-G. Jin Y.-S. Cha Y.-L. Seo J.-H. (2017). Bioethanol production from cellulosic hydrolysates by engineered industrial Saccharomyces cerevisiae. Bioresour Technol. 228, 355–361. doi: 10.1016/j.biortech.2016.12.042, PMID: 28088640

[B53] Lee H.-J. Lim W.-S. Lee J.-W. (2013). Improvement of ethanol fermentation from lignocellulosic hydrolysates by the removal of inhibitors. J. Ind. Eng. Chem. 19, 2010–2015. doi: 10.1016/j.jiec.2013.03.014

[B54] Lee J.-W. Trinh L. T. P. Lee H.-J. (2014). Removal of inhibitors from a hydrolysate of lignocellulosic biomass using electrodialysis. Separation Purification Technol. 122, 242–247. doi: 10.1016/j.seppur.2013.11.008

[B55] Liu W. Liu P. Liu L. Sun H. Fan Y. Ma C.. (2024). Promoting microbial fermentation in lignocellulosic hydrolysates by removal of inhibitors using MTES and PEI-modified chitosan-chitin nanofiber hybrid aerogel. Carbohydr. Polymers 328, 121766. doi: 10.1016/j.carbpol.2023.121766, PMID: 38220334

[B56] Long T. M. Su Y.-K. Headman J. Higbee A. Willis L. B. Jeffries T. W. (2012). Cofermentation of glucose, xylose, and cellobiose by the beetle-associated yeast Spathaspora passalidarum. Appl. Environ. Microbiol. 78, 5492–5500. doi: 10.1128/AEM.00374-12, PMID: 22636012 PMC3406140

[B57] Mahmud M. A. Anannya F. R. (2021). Sugarcane bagasse - A source of cellulosic fiber for diverse applications. Heliyon 7, e07771. doi: 10.1016/j.heliyon.2021.e07771, PMID: 34458615 PMC8379461

[B58] Marques W. L. Raghavendran V. Stambuk B. U. Gombert A. K. (2016). Sucrose and Saccharomyces cerevisiae: a relationship most sweet. FEMS Yeast Res. 16, fov107. doi: 10.1093/femsyr/fov107, PMID: 26658003

[B59] Nair L. G. Agrawal K. Verma P. (2022). An overview of sustainable approaches for bioenergy production from agro-industrial wastes. Energy Nexus 6, 100086. doi: 10.1016/j.nexus.2022.100086

[B60] Ndubuisi I. A. Amadi C. O. Nwagu T. N. Murata Y. Ogbonna J. C. (2023). Non-conventional yeast strains: Unexploited resources for effective commercialization of second generation bioethanol. Biotechnol. Adv. 63, 108100. doi: 10.1016/j.bioteChadv.2023.108100, PMID: 36669745

[B61] Nevoigt E. (2008). Progress in metabolic engineering of Saccharomyces cerevisiae. Microbiol. Mol. Biol. Rev. 72, 379–412. doi: 10.1128/MMBR.00025-07, PMID: 18772282 PMC2546860

[B62] Nguyen N. H. Suh S.-O. Marshall C. J. Blackwell M. (2006). Morphological and ecological similarities: wood-boring beetles associated with novel xylose-fermenting yeasts, Spathaspora passalidarum gen. sp. nov. and Candida jeffriesii sp. nov. Mycol Res. 110, 1232–1241. doi: 10.1016/j.mycres.2006.07.002, PMID: 17011177

[B63] Nilvebrant N.-O. Reimann A. Larsson S. Jönsson L. J. (2001). Detoxification of lignocellulose hydrolysates with ion-exchange resins. Appl. Biochem. Biotechnol. 91, 35–49. doi: 10.1385/ABAB:91-93:1-9:35, PMID: 11963864

[B64] Pacheco T. F. MaChado B. R. C. De Morais Júnior W. G. Almeida J. R. M. Gonçalves S. B. (2021). Enhanced tolerance of spathaspora passalidarum to sugarcane bagasse hydrolysate for ethanol production from xylose. Appl. Biochem. Biotechnol. 193, 2182–2197. doi: 10.1007/s12010-021-03544-6, PMID: 33682050

[B65] Palmonari A. Cavallini D. Sniffen C. J. Fernandes L. Holder P. Fagioli L.. (2020). *Short communication:* Characterization of molasses chemical composition. J. Dairy Sci. 103, 6244–6249. doi: 10.3168/jds.2019-17644, PMID: 32331893

[B66] Palmqvist E. Hahn-hägerdal B. (2000). Fermentation of lignocellulosic hydrolysates. I: inhibition and detoxification. Bioresource Technol. 74, 17–24. doi: 10.1016/S0960-8524(99)00160-1

[B67] Parawira W. Tekere M. (2011). Biotechnological strategies to overcome inhibitors in lignocellulose hydrolysates for ethanol production: review. Crit. Rev. Biotechnol. 31, 20–31. doi: 10.3109/07388551003757816, PMID: 20513164

[B68] Pereira I de O. Dos Santos Â. A. Gonçalves D. L. Purificação M. Guimarães N. C. Tramontina R.. (2021). Comparison of Spathaspora passalidarum and recombinant Saccharomyces cerevisiae for integration of first- and second-generation ethanol production. FEMS Yeast Res. 21, foab048. doi: 10.1093/femsyr/foab048, PMID: 34477865

[B69] Persson S. Shashkova S. Österberg L. Cvijovic M. (2022). Modelling of glucose repression signalling in yeast Saccharomyces cerevisiae. FEMS Yeast Res. 22, foac012. doi: 10.1093/femsyr/foac012, PMID: 35238938 PMC8916112

[B70] Radecka D. Mukherjee V. Mateo R. Q. Stojiljkovic M. Foulquié-Moreno M. R. Thevelein J. M. (2015). Looking beyond Saccharomyces: the potential of non-conventional yeast species for desirable traits in bioethanol fermentation. FEMS Yeast Res. 15, fov053. doi: 10.1093/femsyr/fov053, PMID: 26126524

[B71] Ribeiro L. E. Albuini F. M. Castro A. G. Campos V. J. de Souza G. B. Mendonça J. G. P.. (2021). Influence of glucose on xylose metabolization by Spathaspora passalidarum. Fungal Genet. Biol. 157, 103624. doi: 10.1016/j.fgb.2021.103624, PMID: 34536506

[B72] Rodrussamee N. Sattayawat P. Yamada M. (2018). Highly efficient conversion of xylose to ethanol without glucose repression by newly isolated thermotolerant Spathaspora passalidarum CMUWF1-2. BMC Microbiol. 18, 73. doi: 10.1186/s12866-018-1218-4, PMID: 30005621 PMC6043994

[B73] Roukas T. Kotzekidou P. (2022). From food industry wastes to second generation bioethanol: a review. Rev. Environ. Sci. Biotechnol. 21, 299–329. doi: 10.1007/s11157-021-09606-9

[B74] Saengphing T. Sattayawat P. Kalawil T. Suwannarach N. Kumla J. Yamada M.. (2024). Improving furfural tolerance in a xylose-fermenting yeast Spathaspora passalidarum CMUWF1–2 via adaptive laboratory evolution. Microbial Cell Factories 23, 80. doi: 10.1186/s12934-024-02352-x, PMID: 38481222 PMC10936021

[B75] Saha B. Sakakibara Y. Cotta M. (2007). Production of d-arabitol by a newly isolated Zygosaccharomyces rouxii. J. Ind. Microbiol. Biotechnol. 34, 519–523. doi: 10.1007/s10295-007-0211-y, PMID: 17357803

[B76] Sánchez-Fresneda R. Guirao-Abad J. P. Argüelles A. González-Párraga P. Valentín E. Argüelles J.-C. (2013). Specific stress-induced storage of trehalose, glycerol and D-arabitol in response to oxidative and osmotic stress in Candida albicans. Biochem. Biophys. Res. Commun. 430, 1334–1339. doi: 10.1016/j.bbrc.2012.10.118, PMID: 23261427

[B77] Simpson-Lavy K. Kupiec M. (2019). Carbon catabolite repression in yeast is not limited to glucose. Sci. Rep. 9, 6491. doi: 10.1038/s41598-019-43032-w, PMID: 31019232 PMC6482301

[B78] Singh B. Kumar P. Yadav A. Datta S. (2019). Degradation of fermentation inhibitors from lignocellulosic hydrolysate liquor using immobilized bacterium, *Bordetella* sp. BTIITR. Chem. Eng. J. 361, 1152–1160. doi: 10.1016/j.cej.2018.12.168

[B79] Soares L. B. Bonan C. I. D. G. Biazi L. E. Dionísio S. R. Bonatelli M. L. Andrade A. L. D.. (2020). Investigation of hemicellulosic hydrolysate inhibitor resistance and fermentation strategies to overcome inhibition in non-saccharomyces species. Biomass Bioenergy 137, 105549. doi: 10.1016/j.biombioe.2020.105549

[B80] Subtil T. Boles E. (2012). Competition between pentoses and glucose during uptake and catabolism in recombinant Saccharomyces cerevisiae. Biotechnol. Biofuels 5, 14. doi: 10.1186/1754-6834-5-14, PMID: 22424089 PMC3364893

[B81] Trichez D. Steindorff A. S. de Morais Júnior W. G. Vilela N. Bergmann J. C. Formighieri E. F.. (2023). Identification of traits to improve co-assimilation of glucose and xylose by adaptive evolution of Spathaspora passalidarum and Scheffersomyces stipitis yeasts. Appl. Microbiol. Biotechnol. 107, 1143–1157. doi: 10.1007/s00253-023-12362-1, PMID: 36625916

[B82] Wierckx N. Koopman F. Bandounas L. de Winde J. D. Ruijssenaars H. (2010). Isolation and characterization of Cupriavidus basilensis HMF14 for biological removal of inhibitors from lignocellulosic hydrolysate. Microbial Biotechnol. 3, 336–343. doi: 10.1111/j.1751-7915.2009.00158.x, PMID: 21255332 PMC3815375

[B83] Yang L. Kong W. Yang W. Li D. Zhao S. Wu Y.. (2021). High D-arabitol production with osmotic pressure control fed-batch fermentation by *Yarrowia lipolytica* and proteomic analysis under nitrogen source perturbation. Enzyme Microbial Technol. 152, 109936. doi: 10.1016/j.enzmictec.2021.109936, PMID: 34715526

[B84] Ye S. Kim J.-W. Kim S. R. (2019). Metabolic engineering for improved fermentation of L-arabinose. J. Microbiol. Biotechnol. 29, 339–346. doi: 10.4014/jmb.1812.12015, PMID: 30786700

[B85] Zhao Z. Xian M. Liu M. Zhao G. (2020). Biochemical routes for uptake and conversion of xylose by microorganisms. Biotechnol. Biofuels 13, 21. doi: 10.1186/s13068-020-1662-x, PMID: 32021652 PMC6995148

[B86] Zou J. Chang X. (2022). Past, present, and future perspectives on whey as a promising feedstock for bioethanol production by yeast. J. Fungi (Basel) 8, 395. doi: 10.3390/jof8040395, PMID: 35448626 PMC9031875

