# Supplementary material for: New insights into carbon metabolism in Spathaspora passalidarum for second-generation ethanol production
Source: Front Fungal Biol. 2025 Sep 19;6:1657121. doi: 10.3389/ffunb.2025.1657121 (PMC12491191; doi:10.3389/ffunb.2025.1657121)
Supplement: Supplementary file 1 [file Supplementaryfile1.docx]

**New Insights into Carbon Metabolism in Spathaspora passalidarum for Second-Generation Ethanol Production**

Sofía Racca ^a, b^, Rodrigo J. Leonardi ^a,c^ , Raúl N. Comelli ^a, c, (*)^

| **Agro-industrial Waste** | **Unit** | **Sugar composition** | **Reference** |
| --- | --- | --- | --- |
| *Brewery Spent Grain (BSG)* | g/Kg | Lignin (119-278) | (23) |
|  |  | Celullose (168-253) |  |
|  |  | Xylose (136-209) |  |
|  |  | Arabinose (56-90) |  |
| *Sugarcane bagasse* | %DM | Lignin (19-24) | (24; 25) |
|  |  | Celullose (32-48) |  |
|  |  | Hemicelullose (27-32) |  |
| *High Fructose Syrup (HFS)* | % (w/V) | 42 % HFS: Frutose (43); Dextrose (52) | (26) |
|  |  | 55% HFS: Fructose (55); Dextrose (42) |  |
|  |  | 90% HSF: Fructose (90); Dextrose (7) |  |
| *Beer wort* | %DM | Maltose (60) | (27) |
|  |  | Fructose (2) |  |
|  |  | Glucose (20) |  |
|  |  | Sucrose (3) |  |
|  |  | Maltotriose (10) |  |
| *Fuit juice industry wastes* | %DM | 794 +/- 20 (mg sugar/ Kg waste) | (28) |
|  |  | Fructose (79) |  |
|  |  | Glucose (16) |  |
|  |  | Arabinose (3) |  |
|  |  | Xylose (1) |  |
|  |  | Sucrose (1) |  |
| *Whey permeate* | % (w/V) | Lactose 4,5-5 | (29) |
| *Wheat industry wastes* | %DM | Starch (11) | (30) |
|  |  | Celullose (10,7) |  |
|  |  | Hemicelullose (39) |  |
|  |  | Ligning (5) |  |
| *Can molasse* | %DM | Sucrose (48,8) | (30;31) |
|  |  | Glucose (5,29) |  |
|  |  | Fructose (8,07) |  |
|  |  | Galactose (0,04) |  |
|  |  | Arabinose (0,01) |  |
| *Beet molasse* | %DM | Sucrose (60,9) | (30;31) |
|  |  | Glucose (0,28) |  |
|  |  | Fructose (0,29) |  |
| *Soft drink waste* | % (w/V) | Glucose, fructose and sucrose (6-18) | (32;33) |
| *Coffee wastes* | %VS | Lignin (5-32) | (34) |
|  |  | Celullose (32-45) |  |
|  |  | Hemicelullose (1-20) |  |
| *Soybean hull* | %DM | Lignin (2,1-13,1) | (4;35) |
|  |  | Celullose (28,6-52,3) |  |
|  |  | Hemicellulose (3,1-33) |  |
| *Rice hull* | %DM | Lignin (15-25) | (36) |
|  |  | Celullose (30-40) |  |
|  |  | Hemicelullose (15-25) |  |

**Figure S1. Agro-industrial waste and their composition**


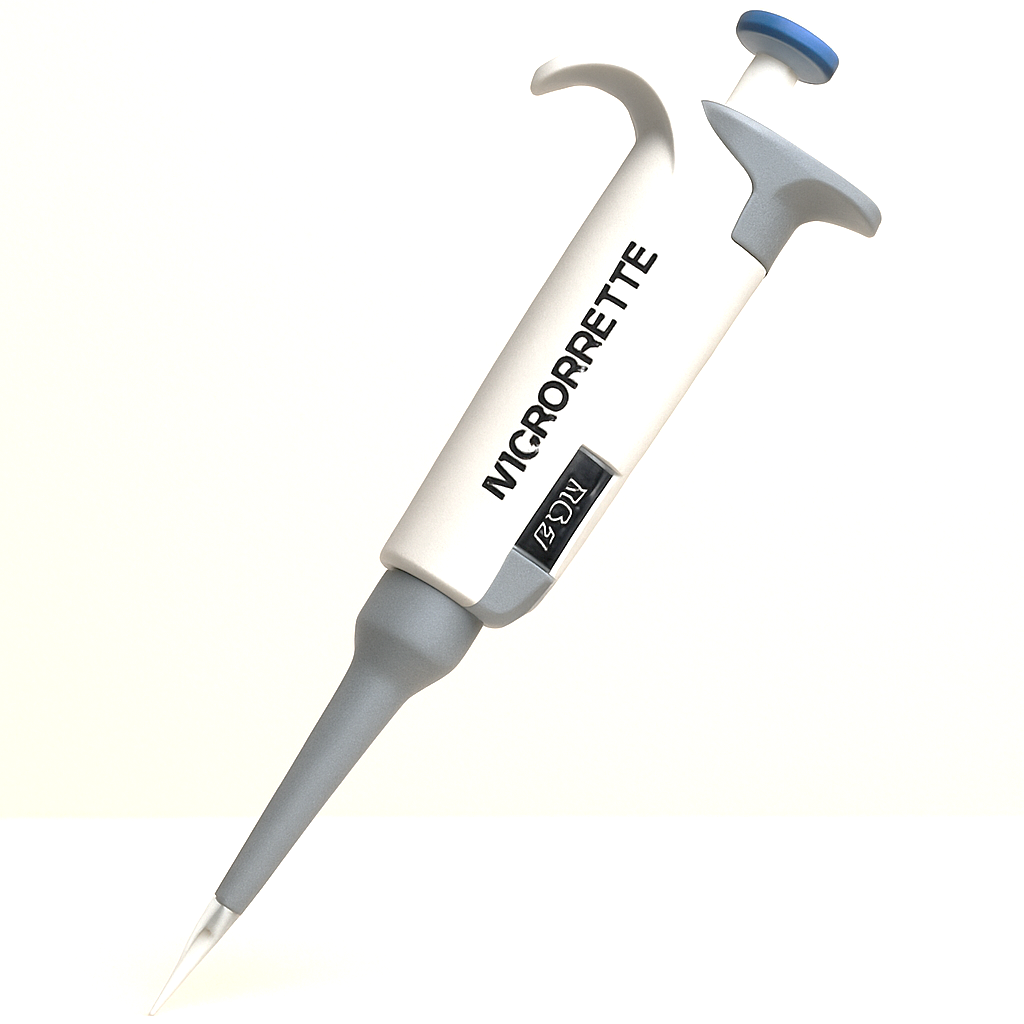

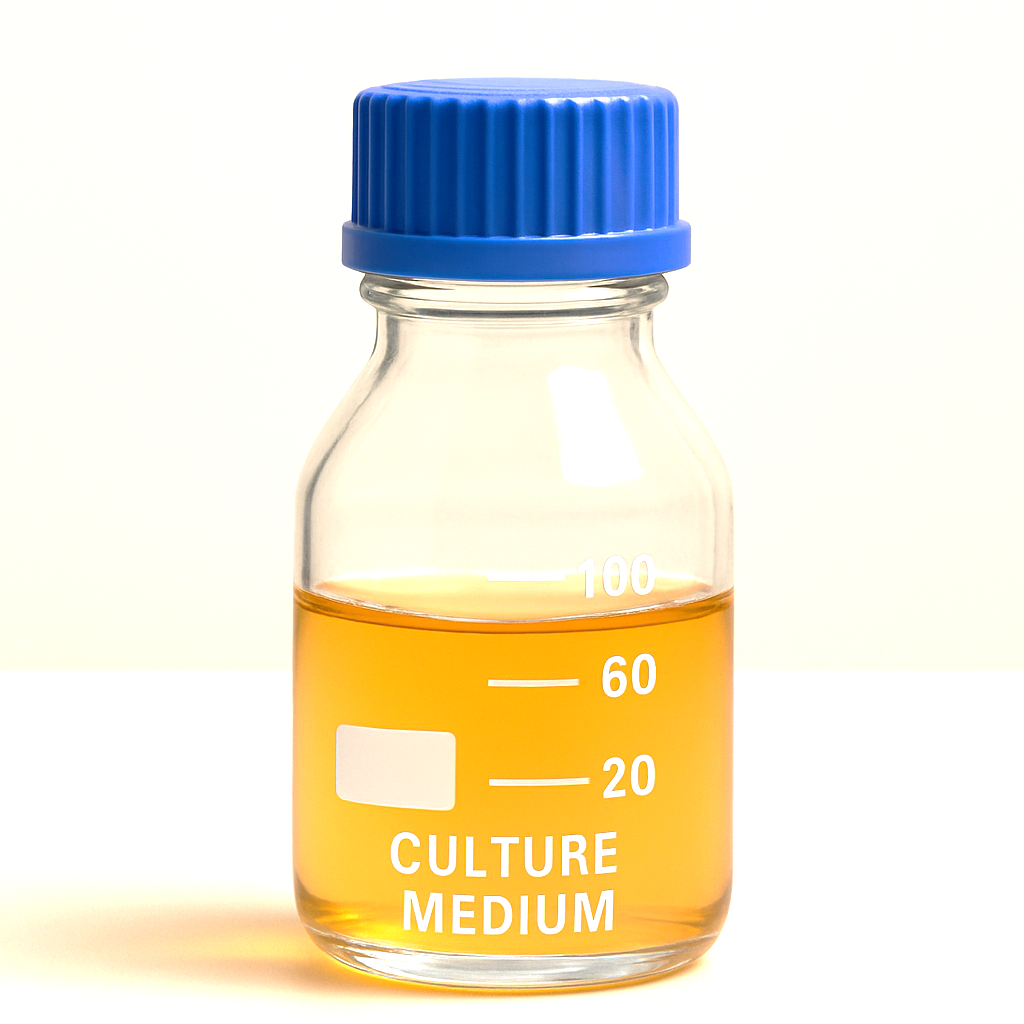

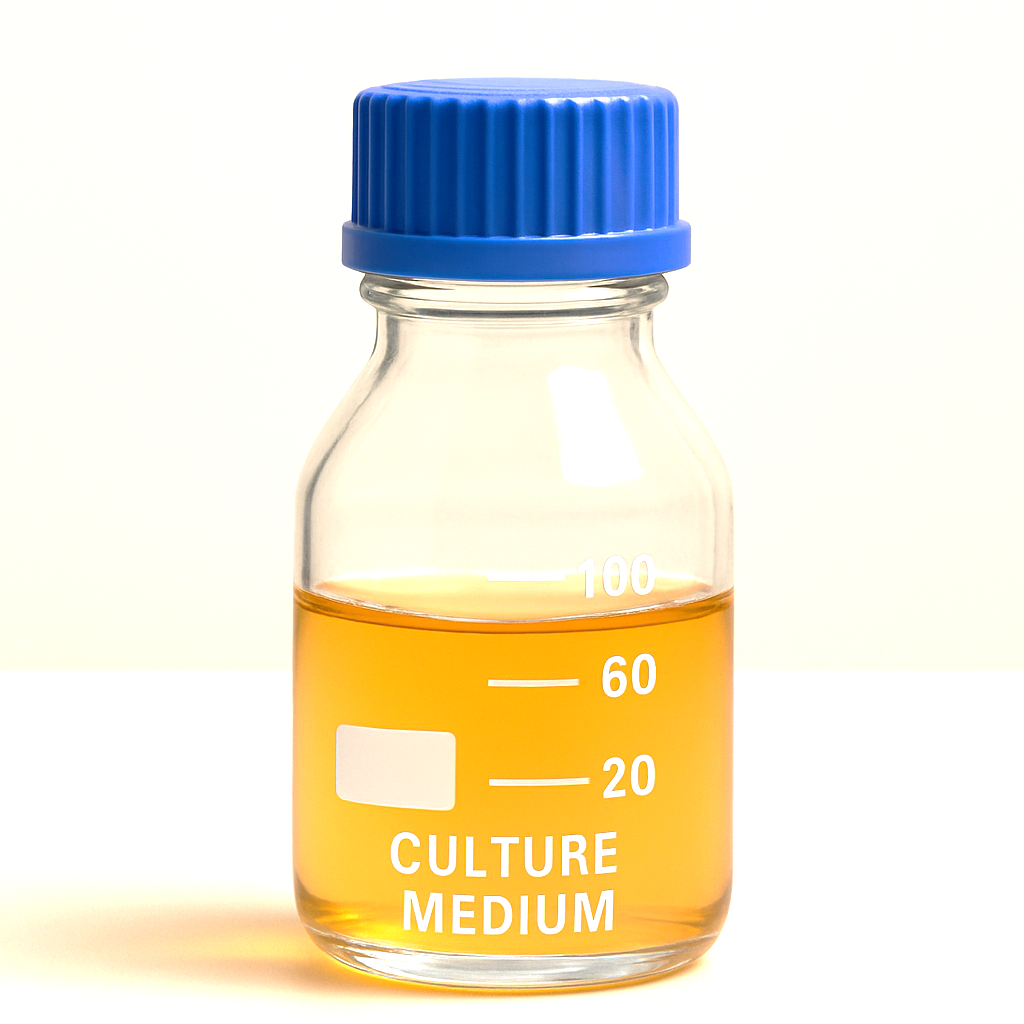


Xylose 20 g L^-1^

Xylose 20 g L^-1^


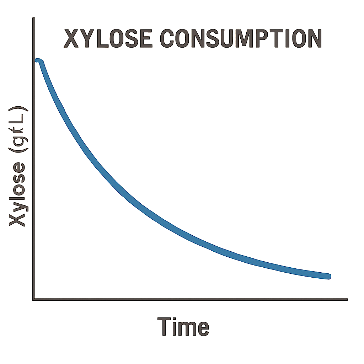

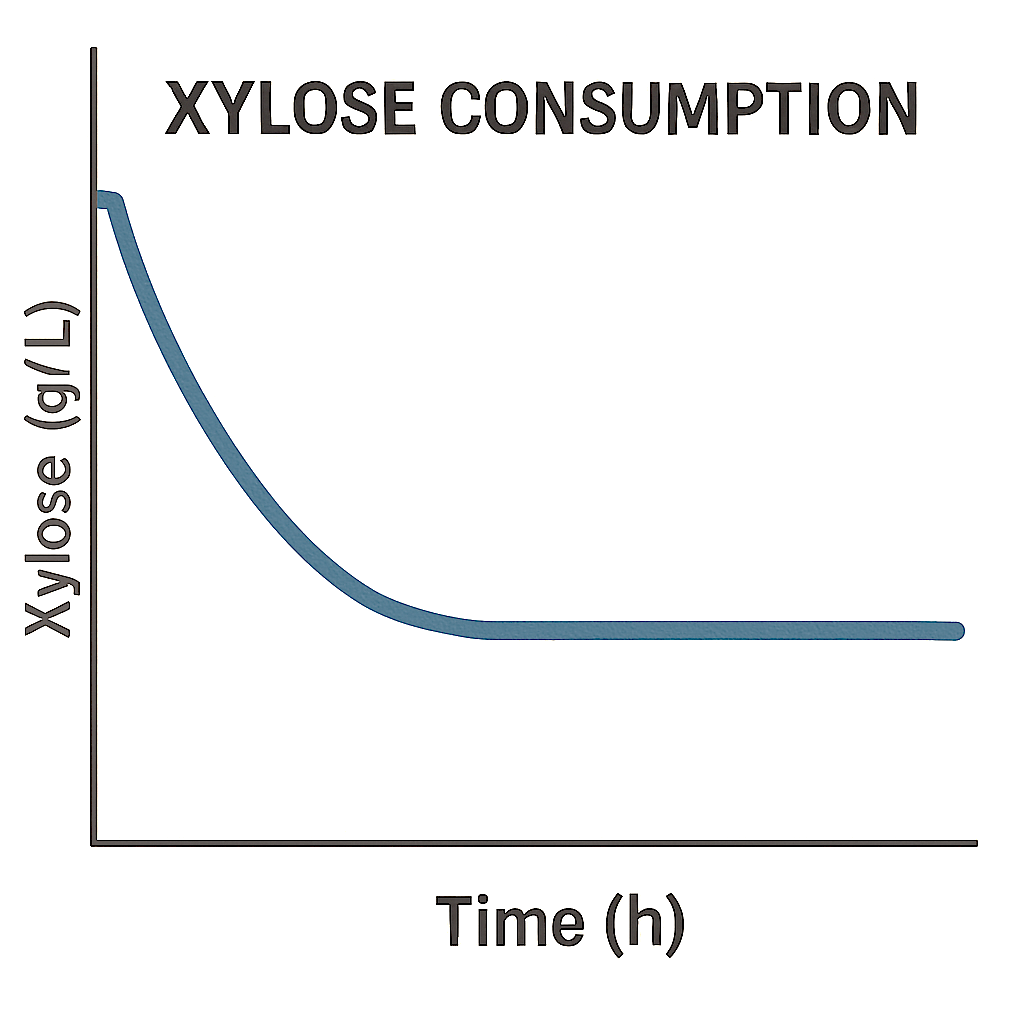


*Could a 12h - Sugar pulse delay xylose metabolism?*

15 g L^-1^

***Sugar Pulse***

after 12 h of fermentation

**Figure S2. Scheme of sugar-pulse methodology during xylose fermentation assays.**

**
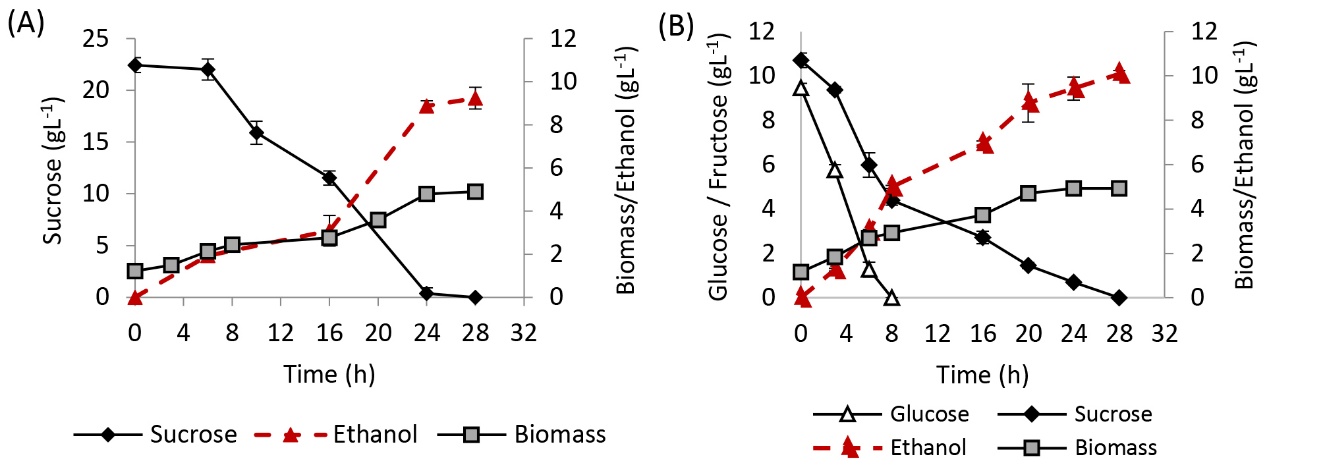
**

**Figure S3. Fermentations were carried out using 20 gL^-1^ of sucrose (a) or 10 gL^-1^ of glucose and fructose (b).** Sugar consumption and biomass or ethanol production are shown.

**
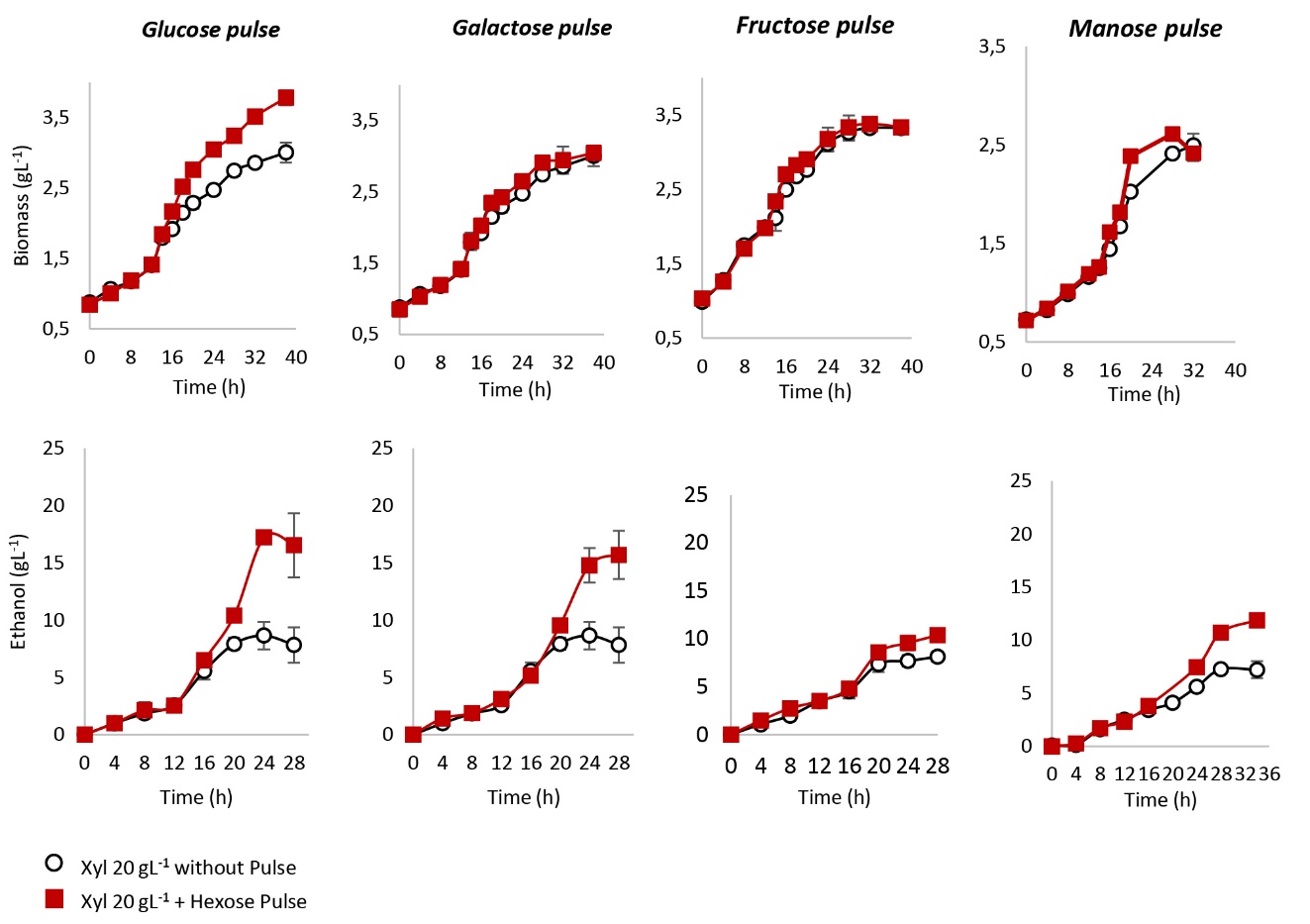
**

**Figure S4.** ***Sp. Passalidarum* fermentation performance in hexose pulse assays: biomass and ethanol quantification.** Fermentations were carried out in 100ml-batch reactors with 60 ml of YPX medium interrupted with a hexose pulse. Values registered in reactors with xylose and hexose pulses are indicated with red squares. Black circles shows biomass and ethanol quantifications in YPX control (30° and 150 rmp).


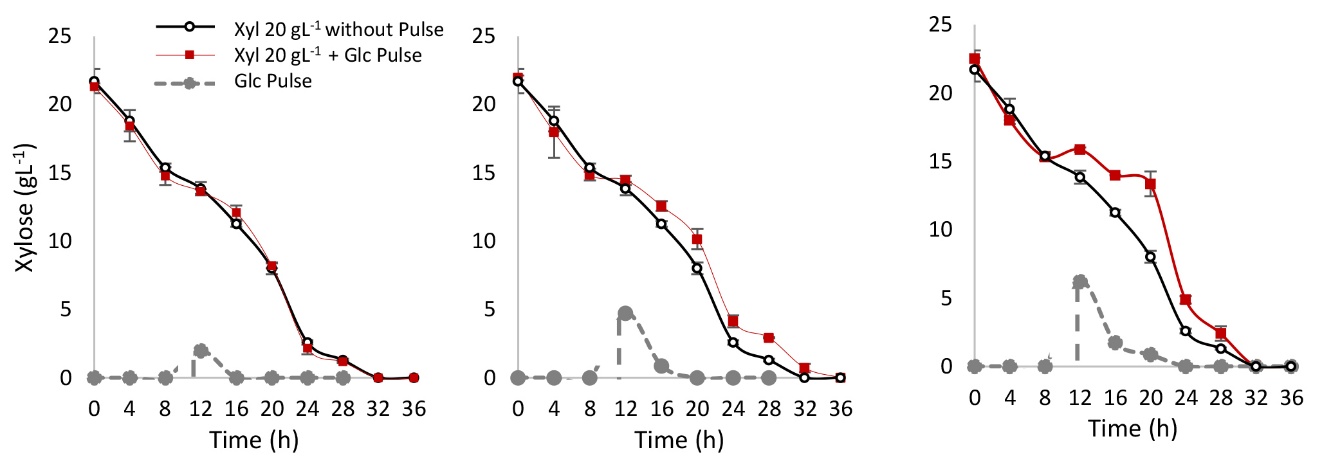


**Figure S5. Dose dependent inhibitory effect of glucose on xylose metabolism.** Fermentations were carried out in 100ml-batch reactors with 60 ml of YPX medium interrupted with glucose pulses of 2, 5 and 7 gL-^1^ . Red squares shows xylose consumption pattern in reactors with pulse assays, while black circles shows xylose quantification in YPX control without pulse.


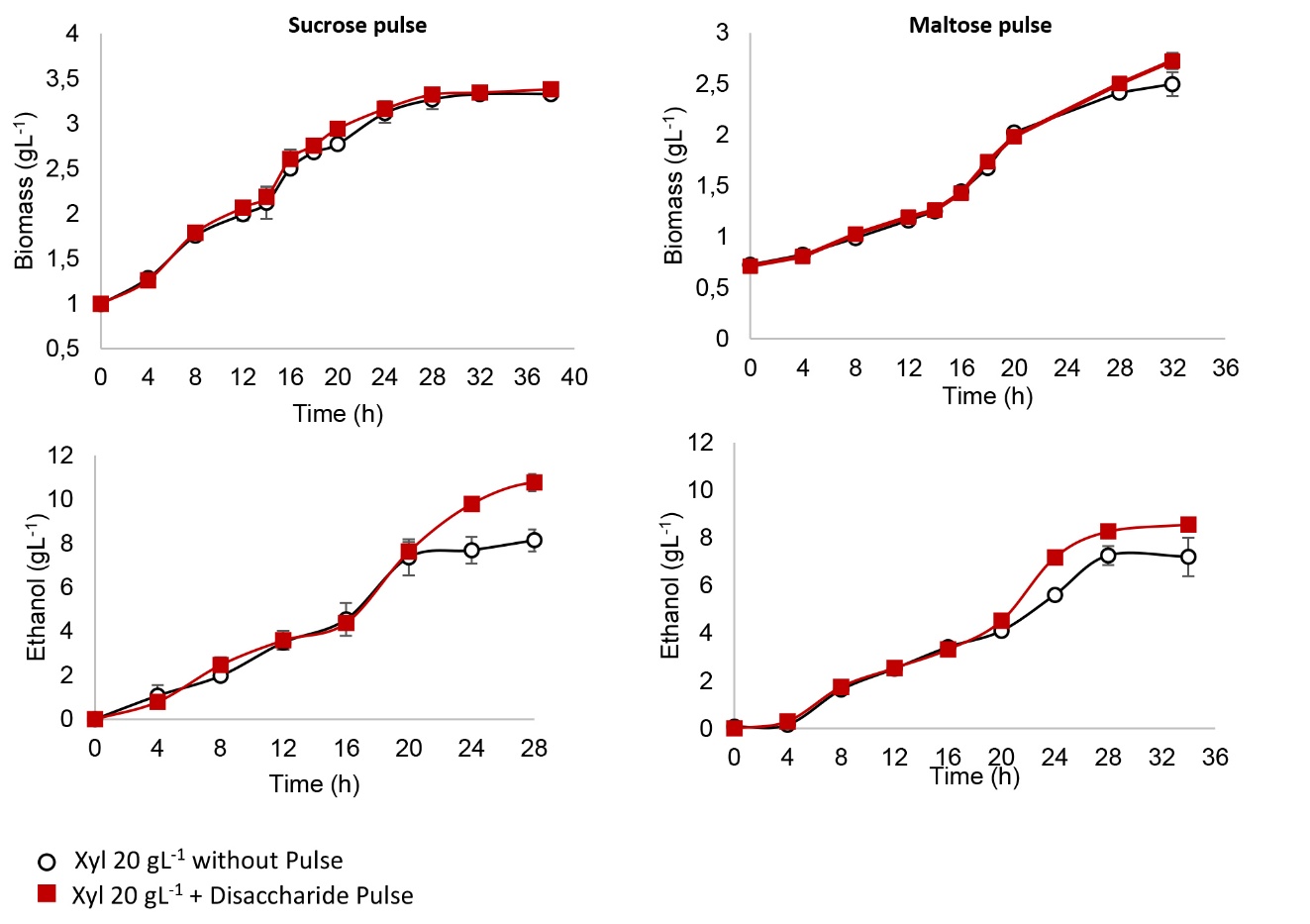


**Figure S6. *Sp. Passalidarum* fermentation performance in disaccharide pulse assays: biomass and ethanol quantification.** Fermentations were carried out in 100ml-batch reactors with 60 ml of YPX medium interrupted with a disaccharide pulse. Values registered in reactors with xylose and disaccharides pulses are indicated with red squares. Black circles shows biomass and ethanol quantifications in YPX control (30° and 150 rmp).

***Figure S7. S. passalidarum performance using a 4-sugar culture media, composed by 15 g L^-1^ of xylose and 5 g L^-1^ of glucose, fructose and maltose.***


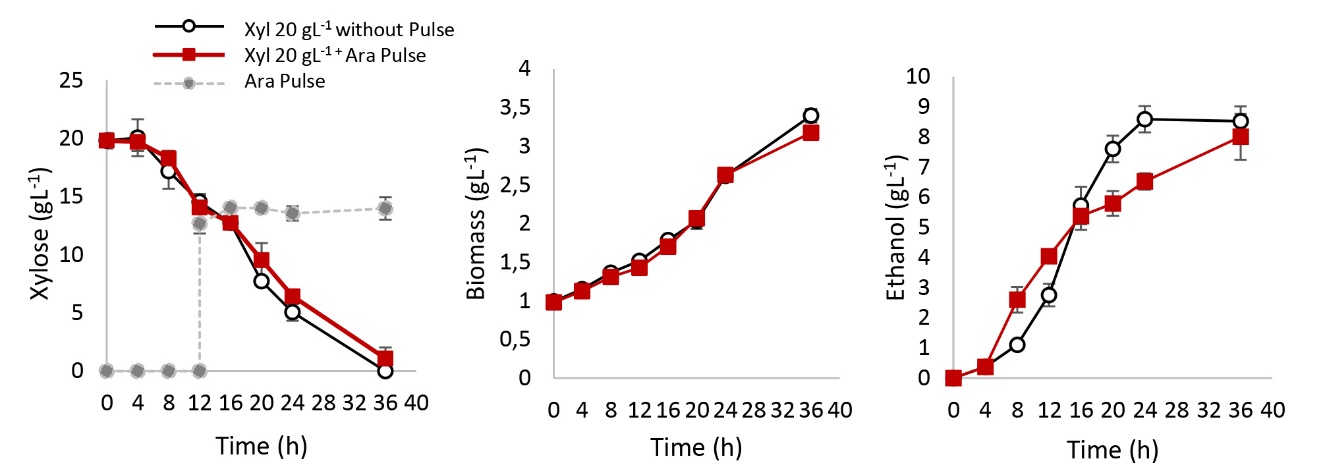


**Figure S8. *Sp. Passalidarum* fermentation performance in YPX reactors interrupted with arabinose pulse.** Fermentations were carried out in 100ml-batch reactors with 60 ml of YPX medium interrupted with an arabinose pulse. Xylose, biomass and ethanol graphs over time are displayed. Red squares indicates values registered in YPX reactors with arabinose pulse; Back circles indicates values registered in YPX control reactors without pulses.
